# Human Organotypic Airway and Lung Organoid Cells of Bronchiolar and Alveolar Differentiation Are Permissive to Infection by Influenza and SARS-CoV-2 Respiratory Virus

**DOI:** 10.3389/fcimb.2022.841447

**Published:** 2022-03-14

**Authors:** Camilla Tvedt Ekanger, Fan Zhou, Dana Bohan, Maria Lie Lotsberg, Maria Ramnefjell, Laurence Hoareau, Gro Vatne Røsland, Ning Lu, Marianne Aanerud, Fabian Gärtner, Pirjo Riitta Salminen, Mariann Bentsen, Thomas Halvorsen, Helge Ræder, Lars A. Akslen, Nina Langeland, Rebecca Cox, Wendy Maury, Linda Elin Birkhaug Stuhr, James B. Lorens, Agnete S. T. Engelsen

**Affiliations:** ^1^ Department of Biomedicine, Faculty of Medicine, University of Bergen, Bergen, Norway; ^2^ Centre for Cancer Biomarkers, University of Bergen (CCBIO), Department of Clinical Medicine, Bergen, Norway; ^3^ The Influenza Centre, Department of Clinical Science, University of Bergen, Bergen, Norway; ^4^ Department of Microbiology and Immunology, University of Iowa, Iowa City, IA, United States; ^5^ Department of Pathology, Haukeland University Hospital, Bergen, Norway; ^6^ Department of Clinical Science, Faculty of Medicine, University of Bergen, Bergen, Norway; ^7^ Department of Pediatrics, Haukeland University Hospital, Bergen, Norway; ^8^ Department of Thoracic Medicine, Haukeland University Hospital, Bergen, Norway; ^9^ Section of Cardiothoracic Surgery, Department of Heart Disease, Haukeland University Hospital, Bergen, Norway; ^10^ Department of Medicine, Haukeland University Hospital, Bergen, Norway; ^11^ Department of Microbiology, Haukeland University Hospital, Bergen, Norway

**Keywords:** human airway and lung organoid model, respiratory epithelium, regional differences, histopathology, viral infection and replication, influenza virus, SARS-CoV-2, pandemic preparedness

## Abstract

The ongoing coronavirus disease 2019 (COVID-19) pandemic has led to the initiation of unprecedented research efforts to understand the pathogenesis mediated by Severe Acute Respiratory Syndrome Coronavirus 2 (SARS-CoV-2). More knowledge is needed regarding the cell type-specific cytopathology and its impact on cellular tropism. Furthermore, the impact of novel SARS-CoV-2 mutations on cellular tropism, alternative routes of entry, the impact of co-infections, and virus replication kinetics along the respiratory tract remains to be explored in improved models. Most applied virology models are not well suited to address the remaining questions, as they do not recapitulate the histoarchitecture and cellular composition of human respiratory tissues. The overall aim of this work was to establish from single biopsy specimens, a human adult stem cell-derived organoid model representing the upper respiratory airways and lungs and explore the applicability of this model to study respiratory virus infection. First, we characterized the organoid model with respect to growth pattern and histoarchitecture, cellular composition, and functional characteristics. Next, *in situ* expression of viral entry receptors, including influenza virus-relevant sialic acids and SARS-CoV-2 entry receptor ACE2 and TMPRSS2, were confirmed in organoids of bronchiolar and alveolar differentiation. We further showed successful infection by pseudotype influenza A H7N1 and H5N1 virus, and the ability of the model to support viral replication of influenza A H7N1 virus. Finally, successful infection and replication of a clinical isolate of SARS-CoV-2 were confirmed in the organoids by TCID50 assay and immunostaining to detect intracellular SARS-CoV-2 specific nucleocapsid and dsRNA. The prominent syncytia formation in organoid tissues following SARS-CoV-2 infection mimics the findings from infected human tissues *in situ*. We conclude that the human organotypic model described here may be particularly useful for virology studies to evaluate regional differences in the host response to infection. The model contains the various cell types along the respiratory tract, expresses respiratory virus entry factors, and supports successful infection and replication of influenza virus and SARS-CoV-2. Thus, the model may serve as a relevant and reliable tool in virology and aid in pandemic preparedness, and efficient evaluation of antiviral strategies.

## Introduction

The emergence of novel viruses and virus mutations is inevitable and will only become a greater concern as the global population increases and areas get more densely populated (Arif and Sengupta, 2020; Whiting, 2020). Therefore, it is fundamental to establish and characterize robust pre-clinical models that can be applied instantly to study the virus-host interactions, to learn more about how emerging viruses transmit and interact with their human hosts to mediate human disease. Improved pre-clinical models may also be a useful tool for more reliable preclinical screening of potential prophylactic and therapeutic interventions, and improved selection of promising drug candidates for testing in clinical trials.

Respiratory viral infections are a leading cause of disease and mortality worldwide. Influenza viruses are negative-sense single-stranded RNA viruses of the family, *Orthomyxoviridae* (Bouvier and Palese, 2008). They are amongst the most common human respiratory viruses. According to the World Health Organization (WHO), seasonal influenza epidemics are estimated to result in about 3 to 5 million cases of severe illness and 290,000 to 650,000 deaths annually (World Health Organization, 2017). Another respiratory virus, severe acute respiratory syndrome coronavirus 2 (SARS-CoV-2), the virus responsible for the ongoing COVID-19 pandemic, belongs to the virus family *Coronaviridae*, and is an enveloped positive-sense single-stranded RNA virus (Zhu et al., 2020). The SARS-CoV-2 virus spread rapidly across countries and the COVID-19 outbreak was declared a pandemic by the World Health Organization (WHO) on March 11^th^ 2020 (World Health Organization, 2020). As of 30 January 2022, over 370 million confirmed COVID-19 cases and more than 5.6 million deaths have been reported to World Health Organization (WHO) (World Health Organization, 2022). The mortality is primarily linked to acute respiratory distress syndrome (ARDS) (Huang et al., 2020) and the long-term effects of infection are still not known, highlighting the urgent need for rapid and extensive scientific research to limit further impact on societies and public health. Despite the intense research efforts since the outbreak of the ongoing COVID-19 pandemic, there are still a lot of uncertainties related to virus replication, pathogenesis, the long-term effects of COVID-19 and the significance of novel SARS-CoV-2 mutations.

Animal models have greatly contributed to our understanding of disease mechanisms and still constitute important pre-clinical tools that allows us to study *in vivo* replication of viruses and the viral interaction with the animal host defense system. However, biological processes occurring in humans may not always be faithfully reproduced in animal models, not even in non-human primate models, and this limits the applicability of the knowledge derived from animal models. One of the particular issues for virology is that several animal models that allow infection of human viruses rely on ectopic expression of entry factors, this implies that the expression pattern and the transcriptional regulation of viral entry factor expression is not identical to the human situation (van der Vaart et al., 2021; Yinda et al., 2021). Further, in virology, monolayers, also known as 2D cell cultures, of animal cells such as the African green monkey kidney cell line, VeroE6 and the canine kidney cell line, MDCK, are highly susceptible to infection by certain viruses and frequently applied to study viral replication and spread (Lugovtsev et al., 2013; Matsuyama et al., 2020). The human alveolar basal epithelial cell line, A549, derived from a human adenocarcinoma, and other human cancer cell lines, such as the human lung cancer cell line, Calu-3 2B4, are also frequently applied (Giard et al., 1973; Yoshikawa et al., 2010). However, although modified animal cell culture models or human cancer cell lines are optimized to support viral replication, they do not recapitulate the heterogeneity and complexity of human tissues, and not even the intracellular processes of primary cells of the organ they derive from. Since most of these *in vitro* models lack a functional Type 1 interferon response, they are also not suitable to assess how the epithelial cells communicate with the immune cells upon infection. Furthermore, primary human airway epithelial cells (pHAE) cultures are commonly used models for studying respiratory tract biology, respiratory tract diseases and therapeutic interventions. pHAE cultures functionally and morphologically resemble the epithelium of the upper conducting airways *in vivo* (Jonsdottir and Dijkman, 2015) and numerous human coronaviruses have been successfully propagated in pHAE cultures (Dijkman et al., 2013; Kindler et al., 2013). A recent study demonstrated the utility of pHAE cultures to model SARS-CoV-2 infection, and showed that SARS-CoV-2 infection of primary human airway epithelial cultures did not trigger an IFN response, but they are sensitive to the effect of both type I and type III IFNs (Vanderheiden et al., 2020). However, pHAE cultures have a restricted proliferative lifespan in culture, and may lose the ability to produce mucus or form cilia after only 3-4 population doublings (Gray et al., 1996; Martinovich et al., 2017). Improvements in the pHAE culture protocols are continuously being made, and include the culture of pHAE at the air-liquid interface for optimal differentiation (Bukowy-Bieryłło, 2021). pHAE cultures may only be applied to model infection of the upper respiratory tract, and not the alveoli, which are the functional units of the lung.

The human respiratory airways and lungs compose a complex vital organ system that can be divided into upper respiratory tract (nose, nasal cavity, and pharynx), and lower respiratory tract (trachea, bronchial tree, lungs) (Ross, 2019). Most of the conducting airways of the respiratory tree from the nasal cavity to the bronchioles consist of pseudostratified columnar epithelium where each region contains stem cell lineages crucial for maintaining tissue homeostasis and regeneration upon injury (Ross, 2019). The pseudostratified columnar epithelium in the conducting airways mainly consists of basal cells, club cells, goblet cells and ciliated cells. In addition, the bronchiolar epithelium may contain a low number of neuroendocrine cells, ionocytes and tuft cells (Montoro et al., 2018; Zepp and Morrisey, 2019). Basal cells are relatively undifferentiated cells and express the transcription factor Trp-63, and cytokeratins 5 and 14, in addition, basal cells are able to self-renew (Zepp and Morrisey, 2019). A subpopulation of basal cell has been found to function as airway epithelial stem cells that are able to give rise to goblet and multi-ciliated cells (Hong et al., 2004). Ciliated cells play an important role in trapping and removing inhaled pathogens, and motor proteins drive the coordinated directional movement of the beating cilia. Previous studies found that Influenza A virus infection immediately enhance cilia-driven flow and ciliary activity in the airway epithelium (Kamiya et al., 2020). The main secretory cells of the conducting airways include goblet cells and the non-ciliated club cells, also known as bronchiolar exocrine cells and formerly known as clara cells. Mucins such as Muc5AC and Muc5B are synthesized by goblet cells and are stored in vesicles that appear electron-lucent on transmission electron microscopy (TEM) images (Leishangthem et al., 2013). The proteins, Splunc1 and secretoglobins (Scgb1a1, Scgb3a2), are produced by mature club cells and are stored in apical granules that appear electron-dense in TEM images (Barkauskas et al., 2017). Lineage tracing studies have revealed that subpopulations of club cells are able to self-renew and may give rise to ciliated cells as well as goblet cells (Rawlins et al., 2009). Furthermore, studies have also found that Scgb1a1 expressing cells may differentiate to Type 1 pneumocytes (AT1) and Type 2 pneumocytes (AT2) cells (Zheng et al., 2012). More in depth studies of pulmonary pathologies are expected to shed light on this complexity, and the particular marker expression patterns associated with the various club cell phenotypes (Zuo et al., 2020).

In recent years a great amount of research has been allocated to the development of novel organotypic 3D culture models, namely organoids. Organoids are complex 3D multicellular constructs that can be derived from either induced pluripotent stem cells (iPSCs) or adult stem cells and grown in a supportive extracellular matrix (*e.g.* Matrigel) to mimic in particular basement membrane components. Cells grown in this 3D culture system have been found to resemble more closely the *in vivo* environment, where cells spontaneously self-organize into hierarchies of stem/progenitor cells and create a continuum of more differentiated and functional cell types. Several research groups have been successful in the development of adult stem cell derived human airway and lung organoids (Zhou et al., 2018; Sachs et al., 2019; Hoareau et al., 2021), and a long-term expansion culture of the bronchiolar pseudostratified epithelium that contain all major cellular elements was pioneered and described by Hans Clevers’ laboratory (Sachs et al., 2019). However, for unknown reasons, a representative model of human alveolar epithelial cells, the so-called alveolospheres, *in vitro* has proven to be more difficult to establish (Hoareau et al., 2021). The alveoli constitute the functional unit of the lungs and the site where the gas exchange between the air and the bloodstream occurs, and consist of a thin simple squamous epithelium with AT1 and AT2 cells forming the alveolar functional units (Ross, 2019). The lack of appropriate models of the alveoli represents an unmet need in this field. We have recently described a model where adult stem cell derived bronchiolospheres and alveolospheres were derived from the same patient tissue specimen (Hoareau et al., 2021). Here we expand the characterization of this model and assess the potential of this model to support viral infection and replication. Here we aimed to characterize a biologically relevant and reproducible human *in vitro* airway and lung model and subsequently assess its potential as a model for studies of respiratory virus infectivity and virus-mediated pathologies. The main benefit of this model is that bronchiolar and alveolar epithelium may be efficiently differentiated from adult stem cells in a single biopsy specimen, and thus favour downstream analysis of regional differences in the response to viral infection.

The interdisciplinary development and application of human airway and lung organoids in virology holds the potential to significantly aid in our understanding of virus- host interactions, pathogenesis and drug development. Thus, continued collaborative efforts to advance this development is urgently needed to increase global pandemic preparedness. The application of organoid cultures representing the epithelium from the various part of the respiratory tract is of particular importance to allow further insight into the viral tropism (van der Vaart and Clevers, 2021), and also, how various mutants affect cell type specific replication kinetics and communication with the immune system. Here, we present for the first-time a thorough characterization of a reproducible model where adult stem cell derived bronchiolospheres and alveolospheres are derived from single human biopsy specimens and conclude that the model is representative of the bronchiolar epithelium of the conducting airways and the respiratory epithelium of the alveoli, respectively. Furthermore, we confirmed the expression of viral entry factors for respiratory virus, including influenza virus and SARS-CoV-2, and conclude that the model system is easily infectable and well suited for virology studies. We propose that this representative model of human airways and lungs can be easily established in virology laboratories and applied in future studies where regional response to respiratory virus or other pathogens are needed.

## Results

### Morphology and Growth Rate Measurements of the Established Human Airway Organoids of Bronchiolar and Alveolar Differentiation

**Figure 1:**
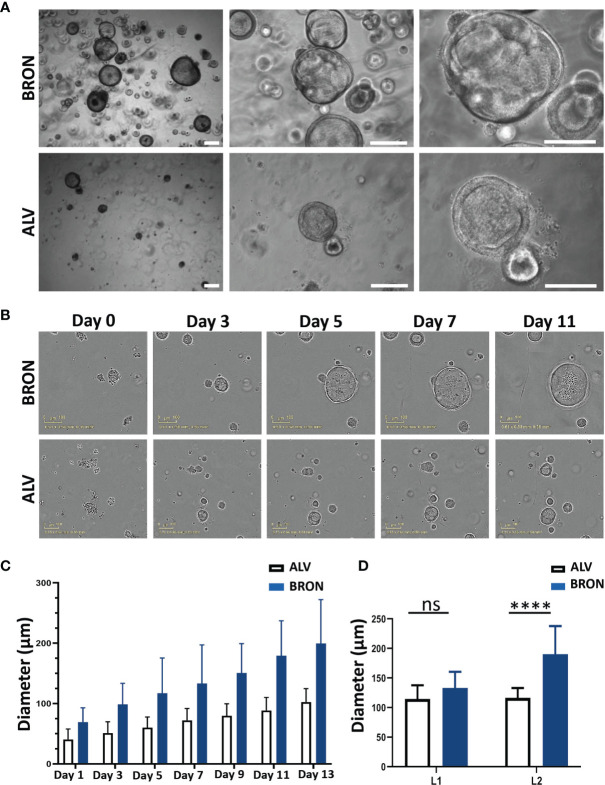
Morphology and growth pattern of established human lung organoids of bronchiolar and alveolar differentiation. **(A)** Representative brightfield images of organoids established in bronchiolar (upper panel) and alveolar (lower panel) differentiation culture media. Magnification, 4x left, 10x middle and 20x right. Scale bars in all images equals 250 µm. The representative images displayed here are from organoids derived from Patient L1. **(B)** IncuCyte Live Cell analysis system (Sartorius) was applied to assess formation of single cells into alveolar and bronchiolar differentiated organoids for 13 days. Representative images from the IncuCyte time course experiment from day 0 until day 11 is shown. Scale bar equals 100 µm. The representative images displayed here are from organoids derived from Patient L2. Link to time-lapse video showing the organoid formation in alveolar and bronchiolar differentiation media of this IncuCyte time-lapse experiment is available in **Supplementary Materials (VIDEO S1** and **S2)**. **(C)** IncuCyte images of organoids from panel B were used to measure the average diameter of 10 individual alveolar and bronchiolar organoids, over 13 days, using the built-in measure tool in ImageJ. The 10 largest organoids were measured for each condition. **(D)** Quantification of organoid diameter from H&E-stained images from passage 2 organoids embedded at day 14 after passaging cultured in bronchiolar and alveolar differentiation medium from patients L1 and L2 was performed using the built-in measure tool in ImageJ. The ten biggest organoids were measured for each condition. Representative H&E images from patient L2 are shown in panel B (bronchiolar) and 4B (alveolar). The Mann-Whitney test was used to compare organoid diameter between ALV and BRON (ns in L1, ****P < 0.0001 in L2).

We aimed to establish human airway organoids of bronchiolar and alveolar differentiation based on protocols previously developed and described by our team and others (Sachs et al., 2019; Hoareau et al., 2021). The organoid tissues were supplied with defined media providing the growth factors needed to generate differentiated organoids (**Table 1**). Under optimized conditions, large bronchiolar (BRON, upper panel) and alveolar (ALV, lower panel) differentiated 3D organoids were formed within two weeks, and the organoids displayed a characteristic morphology with a hollow lumen (**Figure 1A**). Furthermore, we aimed to evaluate the growth rate of the organoids, and live cell time lapse imaging revealed that organoids cultured in medium optimized for bronchiolar differentiation, gradually enlarged between day 1 and 11 (**Figure 1B**). At day 13, organoids cultured in medium optimized for bronchiolar differentiation, had an average diameter of ~200 µm. In contrast, organoids in alveolar medium had a smaller average diameter compared to the bronchiolar organoids (~100 µm) (**Figures 1B, C**). Time-lapse videos displaying the growth of alveolar and bronchiolar organoids are available in **Supplementary Materials (VIDEO S1** and **VIDEO S2)**. Upon quantification of organoid diameters from hematoxylin and eosin (H&E) stained formalin fixed and paraffin embedded (FFPE) sections, we could confirm that the trend that organoids of ALV differentiation were smaller than organoids of BRON differentiation for both patients L1 and L2. A significant difference was found only in organoids from patient L2, suggesting that patient specific differences may be expected **(** **Figure 1D** **)**.

**Table 1:** Airway and lung organoid medium, adapted from Sachs et al., and Hoareau et al., (Sachs et al., 2019; Hoareau et al., 2021).

Media component	Supplier	Catalogue number	Final concentration
R-Spondin 1	Peprotech	120-38	500 ng·ml^-1^
FGF 7	Peprotech	100-19	25 ng·ml^-1^
FGF 10	Peprotech	100-26	100 ng·ml^-1^
Noggin	Peprotech	120-10C	100 ng·ml^-1^
A83-01	Tocris	2939	500 nM
Y-27632	Abmole	Y-27632	5 mM
SB202190	Sigma-Aldrich	S7067	500 mM
B27 supplement	Gibco	17504-44	1x
N-Acetylcysteine	Sigma-Aldrich	A9165-5g	1.25 mM
Nicotinamide	Sigma-Aldrich	N0636	5 mM
GlutaMax 100x	Invitrogen	12634-034	1x
Hepes	Invitrogen	15630-056	10 mM
Penicillin/Streptomycin	Invitrogen	15140-122	100 U·ml^-1^/100 mg·ml^-1^
Primocin	*In vivo*gen	Ant-pm-1	50 mg·ml^-1^
Advanced DMEM/F12	Invitrogen	12634-034	1x
*CHIR99021	Stemcell technologies	72054	3 µM

*Only in airway organoid alveolar medium.

### Airway Organoids of Bronchiolar Differentiation Are Organotypic as Determined by Phenotypic and Functional Characterization

**Figure 2:**
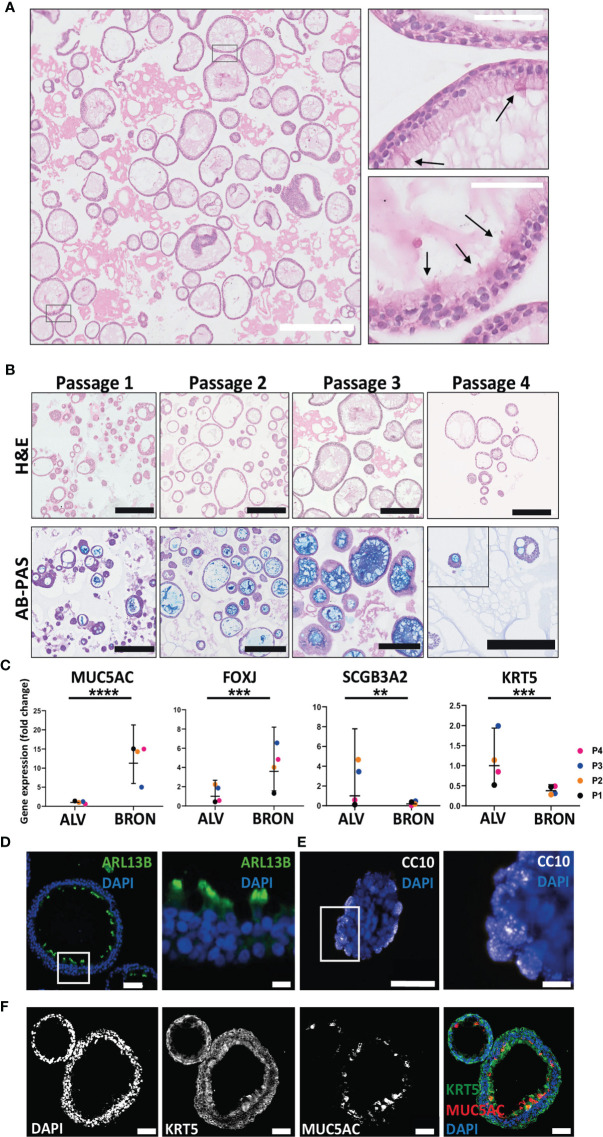
Phenotypic and functional characterization of airway organoids of bronchiolar differentiation (BRON). **(A)** Representative hematoxylin and eosin (H&E) stained formalin fixed paraffin embedded (FFPE) sections display the histology of the organoids of bronchiolar differentiation. Of, note, ciliated cells were observed in the H&E stained FFPE sections (arrows, right zoomed inserts). Scale bar equals 100 µm (left) and 50 µm (right, zoomed inserts). The representative images displayed here are from organoids derived from Patient L2. By microscopy of the organoids in bronchiolar differentiation media in culture, the cilia were found to have a characteristic beating pulse as shown in the **Supplementary Materials video (VIDEO S3)**. **(B)** Images in upper row display the histology of the organoids of bronchiolar differentiation over the four passages (Passage 1- Passage 4). Histology is revealed by H&E-stained formalin fixed paraffin embedded (FFPE) sections. Lower row displays the functional ability of the organoids over the four passages to produce mucus. Mucus production is detected in FFPE sections through alcian blue-Periodic acid schiff (AB-PAS) histochemistry. Positive AB-PAS staining was observed in all passages (P1-P4). Scale bar equals 250 µm in all images. The representative images displayed here are from organoids derived from Patient L2. **(C)** Fold changes in gene expression of BRON compared to ALV organoids. Relative expression of the cell-type markers, MUC5AC (goblet cell), FOXJ1 (ciliated cell), SCGB3A2 (club cell), and KRT5 (basal cell). Relative quantification was performed using the Livac (2^–ΔΔCt^) method. The ALV and BRON organoids have been passaged 4 times (P1-P4) using respective differentiation medium. The mean value of three technical replicates for each passage are shown as individual points. GraphPadPrism v9 was used to calculate and display the geometric mean and 90% confidence interval of the biological replicates (passages). Statistics were performed on the ΔCt values using the Mann-Whitney test. A significant difference in gene expression were detected for MUC5AC (****P < 0.0001), FOXJ (***P = 0.0001), KRT5 (***P = 0.0001) and SCGB3A2 (**P = 0.0080) between alveolar and bronchiolar organoids. The gene expression profile shown here are from patient L2. **(D)** Immunofluorescent staining performed on sections from formalin fixed paraffin embedded (FFPE) tissue of bronchiolar organoids reveal the presence of ciliated cells (ARL13B). Scale bars in all images equals 50 µm. Scale bar zoomed insert equals 10 µm. **(E)** Immunofluorescent staining performed on sections from formalin fixed paraffin embedded (FFPE) tissue of bronchiolar organoids club cells (CC10). Scale bars equals 50 µm for left image and 10 µm for right image (zoom insert). **(F)** Immunofluorescent staining performed on sections from formalin fixed paraffin embedded (FFPE) tissue of bronchiolar organoids confirmed the presence of basal cells (KRT5) and secretory cells (MUC5AC). Scale bars equals 50 µm. The representative images displayed in **(D–F)** are from organoids derived from Patient L2.

Next, we aimed to determine whether the airway organoids of bronchiolar differentiation were organotypic, *i.e.* resembling the tissue of origin with respect to cellular composition, histoarchitecture and functional capabilities. H&E-stained sections of the bronchiolar differentiated organoids revealed that the organoids were composed of a pseudostratified airway epithelium (**Figure 2A**). Ciliated cells were visible in a limited number of sections (**Figure 2A**, zoomed inserts, black arrows). Cilia in motion with the characteristic cilia beat frequency (CBF) of 12 -15 Hz expected in healthy individuals is visible in the cultures, as shown in the video that is available in **Supplementary Materials (VIDEO S3)**. Bronchiolar organoids were passaged every other week for 2 months and dissociated into single cells at the start of each passage. H&E staining of the organoids revealed that the organoids maintained their morphology from passage 1 to passage 4 (**Figure 2B**, upper panel). Alcian-Blue Periodic Acid Schiff (AB-PAS) histochemistry confirmed that the organoids of bronchiolar differentiation efficiently produced mucus throughout the passaging of organoids, Passage 1 to Passage 4 is shown (**Figure 2B**, lower panel). Next, we assayed by RT-qPCR whether the bronchiolar organoid tissues expressed mRNA of genes known to be transcribed in the major cell types of the bronchiolar tissue *in vivo* (**Figure 2C**). Briefly, the marker for goblet cells, MUC5AC, was significantly higher 11.28 fold (****P < 0.0001), in bronchiolar compared to alveolar organoids (**Figure 2C**). The gene expression of FOXJ1, a marker known to be expressed in ciliated cells, displayed a mean fold change upregulation of 3.59 (***P = 0.0001), in bronchiolar organoids compared to alveolar organoids (**Figure 2C** **)**. The markers for club cells and basal cells, SCGB3A2 and KRT5 respectively, displayed a significantly lower expression in bronchiolar organoids compared to alveolar organoids [mean fold change 0.18 (**P = 0.0080) and 0.38 (***P = 0.0001), respectively] (**Figure 2C** **)**. Furthermore, immunofluorescent staining of FFPE tissue sections of the differentiated bronchiolar organoids revealed the presence of all major conducting airway epithelium cell types as expected. Protein markers for the various cell populations, including ciliated cells (ARL13B positive) (**Figure 2D**), club cells (CC10 positive) (**Figure 2E**), goblet cells (MUC5AC positive), and basal cells (KRT5 positive) (**Figure 2F**) were detected in the bronchiolar organoids. The protein-markers, PDPN and SFTPC, which are expected to be expressed in the two main cell types of lung alveoli, namely AT1 and AT2 cells, were detected in moderate levels of expression in bronchiolar organoids. However, the AT2 marker, SFTPC, was not detected in passages 3 and 4 by RT-qPCR (data not shown). Taken, together these results confirm that the applied bronchiolar differentiation protocol induced a representative model of the conducting airway epithelium, with the histoarchitecture, functional abilities, and RNA and protein markers of the various cell types of the tissue expressed as expected.

### Characterization of Bronchiolar Organoid Tissues by Transmission Electron Microscopy (TEM) Reveals Ultrastructural Resemblance to the Airway Respiratory Epithelium

**Figure 3:**
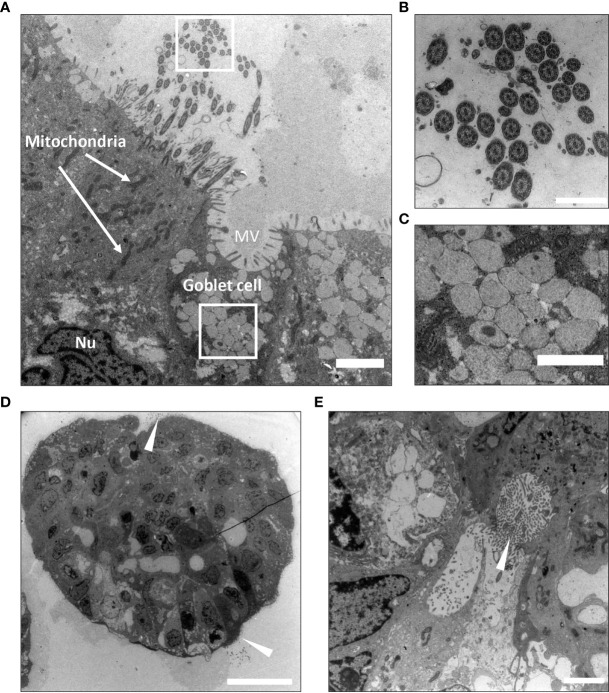
Ultrastructural characterization of bronchiolar organoid tissues by Transmission Electron Microscopy (TEM). **(A)** Transmission electron micrograph display cross-section of a bronchiolar organoid. This image shows the characteristic pseudostratified airway epithelium, consisting of interspersed multi-ciliated and secretory goblet cells. Nu: nucleus. MV: microvilli. Scale bar equals 2000 nm. **(B)** Zoomed insert from **(A)** to show a cross-section of the motile cilia sprouting from the ciliated cell. The characteristic 9 + 2 arrangement of microtubules is clearly visible. Scale equals 500 nm. **(C)** Zoomed insert from **(A)** to show the electron-lucent granules of the secretory goblet cell. Scale bar equals 1000 nm. **(D)** Apical side-out orientated organoids. Ciliated cells are pointed (white arrowheads). Scale bar equals 10 µm. **(E)** Apical side-in orientated organoids. Ciliated cells are pointed (white arrowhead). Scale bar equals 2000 nm. The representative images displayed in these panels **(A–E)** are of organoids derived from Patient L1.

The utility of light microscopy for characterization of cilia are restricted because the diameter of cilia extends to the limit of resolution of this modality and may be further obscured due to fixation artefacts. Thus, although ciliated cells were detected in the H&E stained FFPE sections, transmission electron micrographs better reveal the ultrastructure and demonstrate that a large proportion of the pseudostratified epithelium in the bronchiolar organoids consisted of multi-ciliated cells (**Figure 3**). TEM images also demonstrated the cellular heterogeneity within the organoids, and within the same organoid structure, several cell types were present and a characteristic pseudostratified airway epithelium was revealed (**Figure 3A**). The characteristic 9 + 2 arrangement of microtubules in the cilia is shown in the TEM images (**Figure 3B** **)**. Electron-lucent granules were present in the goblet cells (**Figure 3C**). Of note, some organoids were oriented with the apical side-out (**Figure 3D**), whilst the majority had an apical side-in orientation with the ciliated cells facing the lumen (**Figure 3E**).

### Airway Organoids of Alveolar Differentiation Are Organotypic as Determined by Phenotypic and Functional Characterization

**Figure 4:**
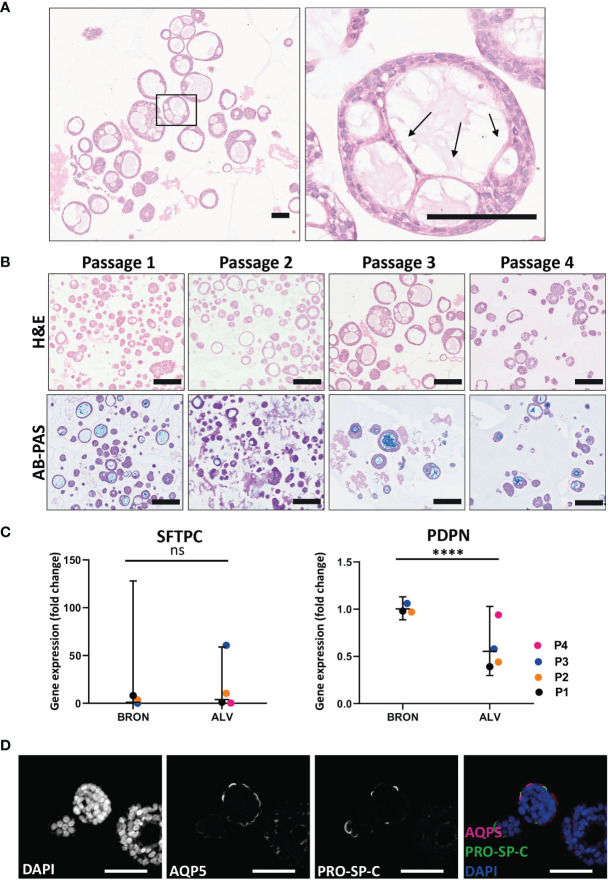
Functional and phenotypic characterization of airway organoids of alveolar differentiation (ALV). **(A)** The morphology of thin-walled cells detected in the H&E-stained alveolar sections resemble the morphology of AT1 cells. Scale bar equals 100 µm. Black arrows point at the thin-walled cells displaying characteristic AT1 morphology. **(B)** Hematoxylin and eosin (H&E) (upper row) and alcian blue-Periodic acid schiff (AB-PAS) (lower row) stained FFPE sections of alveolar organoids over the four passages (Passage 1- Passage 4). Scale bar equals 200 µm. **(C)** Fold changes in gene expression levels of the type 2 pneumocyte (AT2) cell-type marker SFTPC and the type 1 pneumocyte (AT1) cell-type marker PDPN, in the organoids cultured in alveolar medium, that were passaged 4 times (P1-P4). Relative quantification was performed using the Livac method and alveolar organoids are normalized to the average delta Ct of bronchiolar organoids. The mean value of three technical replicates for each passage are shown as individual data points. GraphPadPrism v9 was used to calculate and display the geometric mean and 90% confidence interval of the biological replicates (passages). Statistics were performed on the delta Ct values using the Mann-Whitney test. Expression of SFTPC were 3.90 fold higher in ALV than BRON. (ns). There is a significant difference (fold change 0.55, ****P = 0.0001) in the expression of PDPN gene between the alveolar and bronchiolar organoids, the gene expression profile shown here are from patient L2. **(D)** Immunofluorescent staining of formalin fixed paraffin embedded (FFPE) sections of alveolar organoids showing Aquaporin 5 (AQP5) as a marker for AT1 and pro-surfactant protein C (PRO-SP-C) as a marker for AT2 cells. Scale bar equals 50 µm.

H&E staining of FFPE sections from alveolar differentiation medium was used to assess the histoarchitecture, and elongated, thin-walled cells that resemble AT1 cell morphology were visible (**Figure 4A**, black arrows). The characteristic phenotype was found to be conserved between Passage 1 and Passage 4 (**Figure 4B**, upper panel). Of note, AB-PAS staining to detect mucus production was positive between passage 1 and passage 4 (**Figure 4B**, lower panel). However, by visual inspection of AB-PAS-stained organoid sections, it appeared that fewer cells in the alveolar organoids displayed intracellular AB-PAS staining in the alveolar cultures, compared to the bronchiolar cultures. To assess the presence of the major lung epithelial cells in the alveolar organoids, we analyzed cell specific markers by RT-qPCR and immunofluorescence (**Figures 4C, D** **)**. RT-qPCR revealed that there was no significant difference in the mRNA expression levels of the AT2 cell marker, SFTPC, between bronchiolar and alveolar organoids (Fold change: 3.90, **Figure 4C**). However, a significant difference in PDPN gene expression between bronchiolar and alveolar organoids was detected (Fold change: 0.55, ****P = 0.0001). Albeit at low levels, the RT-qPCR analyses also revealed that the alveolar organoids expressed markers of goblet cells (MUC5AC), ciliated cells (FOXJ1), basal cells (KRT5) and club cells (SCGB3A2), data not shown. The presence of AT1 and AT2 cells *in situ* in the tissue of alveolar organoids was further confirmed by immunostaining of FFPE sections. Immunostaining confirmed the presence of the AT1 marker Aquaporin 5 (AQP5) and AT2 marker pro-surfactant protein C (PRO-SP-C) in the alveolar organoids (**Figure 4D**).

### Organoids of Alveolar and Bronchiolar Differentiation Express Sialic Acid-Containing Receptors

**Figure 5:**
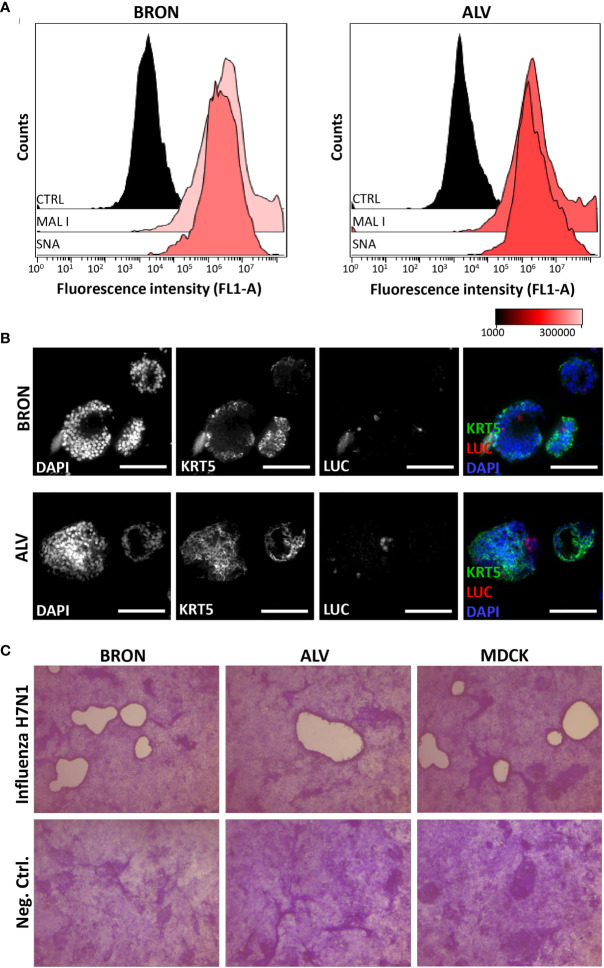
Detection of essential influenza virus receptors entry factors in organoids and infection by influenza pseudotype H5N1 and viral replication by H7N1 virus. **(A)** Sialic acids (SAs) of cell surface glycoproteins and glycolipids are the receptors for influenza virus. The presence of SAs and galactose on bronchiolar (BRON, left histograms) and alveolar (ALV, right histograms) were detected by flow cytometry using the fluorescein-conjugated lectins, MAL I, recognizing galactose, and SNA, recognizing α 2,6-linked sialic acids. CTRL histogram represents the unstained control cells. The representative flow cytometry histograms shown here were from stained dissociated organoids from patient L2. **(B)** *In situ* detection of cells infected by the pseudotyped luciferase expressing H5N1 influenza virus was done by immunostaining to detect luciferase (LUC) and keratin 5 (KRT5) in formalin fixed paraffin embedded (FFPE) sections of infected organoids. Organoids of BRON (upper panel) and ALV (lower panel) organoid cultures are shown. Sections are counterstained with DAPI. Scale bar in all images equals 100 µm. **(C)** Organoids were infected with the replicating influenza virus H7N1 strain at an *estimated* multiplicity of infection (MOI) of 7. Plaque assay was used to confirm efficient replication of pseudotyped influenza virus in the organoid cultures. Infected MDCK cells were used as a positive control in this assay. Plaques are visible in MDCK cell cultures inoculated with conditioned media harvested from infected bronchiolar and alveolar organoid cultures, as well as conditioned media harvested from the positive control MDCK cells. Conditioned media from uninfected controls showed no sign of plaque formation (lower row). Counterstaining by crystal violet. Magnification equals 4x.

Influenza virus entry receptors are known to be expressed in the human airways (Gagneux et al., 2003; Matrosovich et al., 2004), thus, we initially aimed to assess by flow cytometry the expression sialic acid (SA), α 2,6-linked and gal (β-1,4). In particular, assessing α 2,6-linked SA expression is of special interest as it is used by seasonal and highly pathogenic influenza viruses to get an initial foothold in humans (Leung et al., 2012). Gal (β-1,4), recognized by Maackia Amurensis Lectin I (MAL I), and α 2,6-linked SA, recognized by the lectin Sambucus nigra agglutinin (SNA) were shown to be ubiquitously expressed in cells of dissociated organoids of both bronchiolar (**Figure 5A**, left histograms) and alveolar (**Figure 5A**, right histograms) differentiation.

### The Organoids of Bronchiolar and Alveolar Differentiation Support Infection With Pseudotype Influenza H5N1 and H7N1 Viruses

The bronchiolar and alveolar organoids were infected with pseudotyped influenza H5N1 or H7N1 virus that harbor luciferase reporter-gene. First, we verified that transgene expression was dose dependent upon infection with the pseudotyped influenza H7N1 and H5N1 viruses in positive control MDCK cells (data not shown). Next, the bronchiolar and alveolar organoids with endogenous α 2,6-linked SA and galactose expression were infected with pseudotyped influenza H5N1 virus harboring luciferase reporter gene and harvested for FFPE 72 h post-infection. *In situ* detection of infected cells in the alveolar and bronchiolar organoids was performed on the FFPE sections by immunostaining. Infected luciferase positive cells were detected in organoids of bronchiolar (**Figure 5B**, upper row) and alveolar differentiation (**Figure 5B**, lower row). Next, the organoids were infected with a replicating influenza H7N1 virus. The plaque assay was used to confirm efficient replication in the organoids of alveolar and bronchiolar differentiation, with MDCK cells used as a positive control (**Figure 5C** **).**

### Airway Organoids Express Entry Factors Vital for SARS-CoV-2 Infection and Are Permissive to a Clinical Isolate of SARS-CoV-2

**Figure 6:**
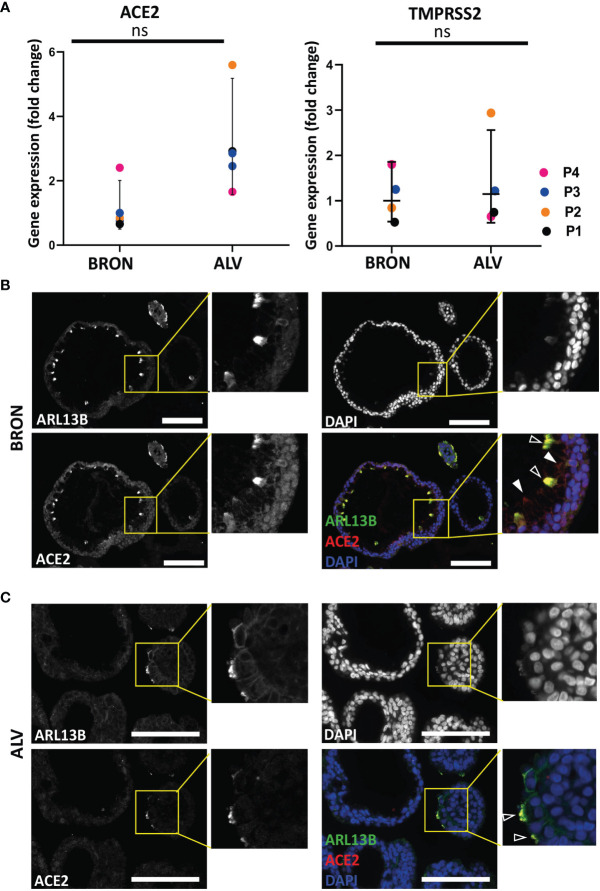
Expression of SARS-CoV-2 entry factors in BRON and ALV organoids. **(A)** ACE2 (left) and TMPRSS2 (right) expression is detected in patient L2 organoids of bronchiolar (BRON) and alveolar (ALV) differentiation from passage 1-4. Relative quantification was performed using the Livac method and alveolar organoids are normalized to the average delta Ct of bronchiolar organoids. The mean value of three technical replicates for each passage are shown as individual data points. GraphPadPrism v9 was used to calculate and display the geometric mean and 90% confidence interval of the biological replicates (passages). Statistics were performed on the delta Ct values using the Mann-Whitney test. No statistically significant differences were observed in the median deltaCt values of ACE2 between BRON and ALV cultures. **(B)** Immunofluorescent staining of formalin fixed paraffin embedded (FFPE) sections of bronchiolar (BRON) organoids (representative images from patient L2 passage 2), display co-staining of the cilia marker (ARL13B) and the SARS-CoV-2 receptor ACE2 (transparent arrowheads). ACE2 staining alone (white arrowheads). Scale bar equals 100 µm. **(C)** Immunofluorescent staining of formalin fixed paraffin embedded (FFPE) sections of alveolar (ALV) organoids (representative images from patient L2 passage 2), display co-staining of the cilia marker (ARL13B) and the SARS-CoV-2 receptor ACE2 (transparent arrowheads). Scale bar equals 100 µm. ns, not significant.

**Figure 7:**
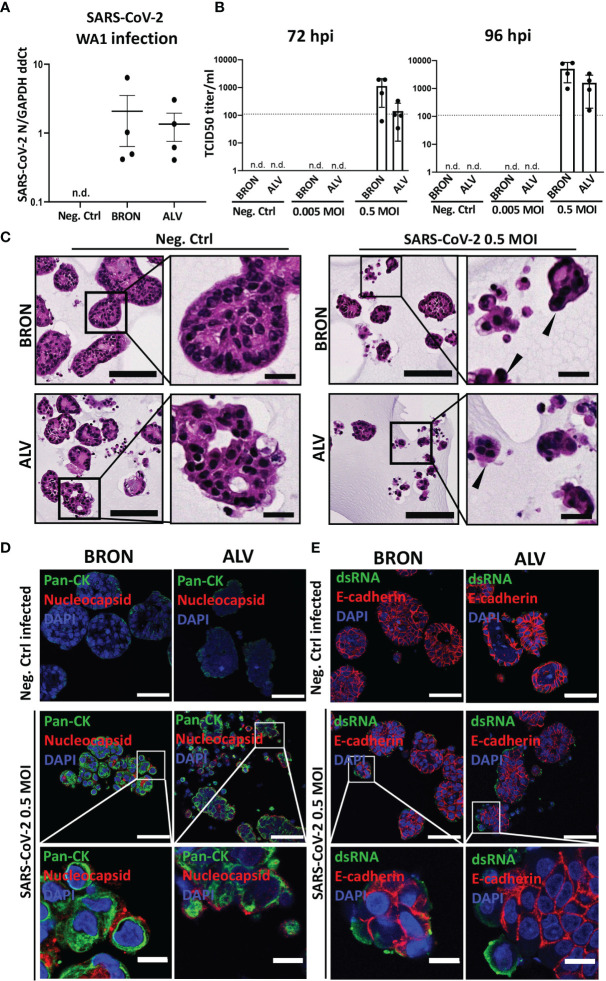
Infection of human BRON and ALV organoid by two clinical isolates (WA-1 and hCoV-19/Norway/Bergen-01/2020) of SARS-CoV-2 virus. **(A)** RT-qPCR was used to detect the expression of the SARS-CoV-2 N gene in bronchiolar organoids infected with a clinical isolate of SARS-CoV-2-WA1 (Washington strain), using the SYBR green method. Data is shown as 2 ^ -(avg Ct SARS-CoV-2 N – avg Ct GAPDH), n.d., not detected. **(B)** Replication of SARS-CoV-2 in organoids of BRON and ALV differentiation was assessed by infection of organoid cultures at multiplicity of infection (MOI) of 0 (Neg.Ctrl), 0.005 or 0.5. Titration of harvested supernatants were performed by TCID50 assay on Vero cells (CCL-81, ATCC). Bar-charts display the quantification of organoid supernatants harvested 72 h post infection (left) and 96 h post infection (right). For each condition the mean +-SD of four biological replicates is shown, n.d., not detected. **(C)** Histopathology of bronchiolar (BRON) and alveolar (ALV) organoid cultures, mock infected (Neg.Ctrl.) or SARS-CoV-2 infected at MOI of 0.5. Samples were harvested 72 hours post infection, and FFPE sections stained by H&E. Formation of multinucleated cells (syncytia, black filled arrowheads) characteristic of SARS-CoV-2 infection in human patient samples were observed in the infected samples. Scale bar in all images equals 100 µm, except zoomed inserts (20 µm). **(D)** *In situ* detection of bronchiolar (BRON, left panel) and alveolar (ALV, right panel) organoid tissue cells infected by mock (Neg.Ctrl.) or 0.5 MOI SARS-CoV-2 virus (hCoV-19/Norway/Bergen-01/2020), was done by immunostaining of FFPE sections to detect SARS-CoV-2 nucleocapsid (red) and pan-cytokeratin (Pan-CK, green). DAPI (blue) nuclear stain. Scale bar in all images equals 50 µm, except lower panel (10 µm). Samples were harvested 72 hours post infection for BRON and 96 hours post infection for ALV. **(E)** Immunofluorescent staining of formalin fixed paraffin embedded (FFPE) sections of bronchiolar (BRON, left panel) and alveolar (ALV, right panel) organoids mock infected (Neg.Ctrl) or infected by SARS-CoV-2 virus (hCoV-19/Norway/Bergen-01/2020), MOI 0.5, (samples were harvested 72 hours post infection for BRON and 96 hours post infection for ALV) display the expression of dsRNA (green) and E-cadherin (red). DAPI (blue) nuclear stain. Scale bar equals 50 µm in all images, except lower panel (10 µm).

Expression of SARS-CoV-2 entry receptor has been shown to be low and restricted to particular cell types in the human airways (Hikmet et al., 2020). In order to determine the expression of the SARS-CoV-2 receptor, ACE2 and serine protease TMPRSS2, we applied gene expression analysis by RT-qPCR, which revealed that bronchiolar and alveolar organoids express ACE2 (**Figure 6A**, left) and TMPRSS2 (**Figure 6A**, right). ACE2 and TMPRSS2 gene expression was also confirmed in organoids derived from an additional patient (**Figure S1B**). Immunofluorescence confirmed the presence of ACE2 in bronchiolar organoids, as shown in **Figure 6B**. Of note, particularly high levels of ACE2 were detected in the ciliated cells, as demonstrated by the colocalization of ARL13B and ACE2. Detection of ACE2 were also shown in the alveolar organoids, as shown in **Figure 6C**. Ultimately, bronchiolar organoids were infected with a clinical isolate of SARS-CoV-2 (2020_WA1), and infection was assessed using RT-qPCR analysis to detect the presence of the SARS-CoV-2 N gene. For the vehicle treated control, no expression of SARS-CoV-2 N gene was detected. However, expression of SARS-CoV-2 N gene was detected in SARS-CoV-2-WA1 infected bronchiolar organoids (**Figure 7A** **)**. Successful infection of alveolar organoids was also detected. This proved that bronchiolar and alveolar organoids are indeed permissive to infection by the clinical isolate SARS-CoV-2 WA1.

In order to rule out the possibility that virus is getting into the organoids (as detected by RT-qPCR) but not producing infectious virus in the model, so called abortive infection, we performed an infectivity assay (TCID50) on Vero cells for supernatants collected from hCoV-19/Norway/Bergen-01/2020 infected organoid cultures at 72 and 96 h post-infection, irrespectively. TrypLE was used to dissociate the organoids, prior to infection, to ensure accessibility of SARS-CoV-2 virus to the cells oriented towards the organoid lumen. The assay showed that organoids of bronchiolar (BRON) and alveolar (ALV) differentiation were able to produce infectious viral particles, and that the viral titer of samples harvested 96 h post-infection increased compared to the viral titer of samples harvested 72 h post-infection, for both BRON and ALV conditions **(** **Figure 7B** **).** Histopathological examination were performed on H&E stained FFPE sections from organoid cultures from infection experiment. Comparison of SARS-CoV-2 infected samples to the mock infected samples (Neg.Ctrl.) revealed characteristic cytopathic effect in the infected samples of both bronchiolar (BRON, **Figure 7C**, upper row) and alveolar (ALV, **Figure 7C**, lower row) differentiation. In particular, the prominent formation of multinucleated cells, also known as syncytia, may indicate virus production in the infected sample. The presence of intracellular SARS-CoV-2 specific nucleocapsid protein in the infected cells further support the notion that ALV and BRON organoid cells were successfully infected by the SARS-CoV-2 virus (**Figure 7D**). The detection of dsRNA in the infected specimens **(** **Figure 7E**) served to support the successful replication of SARS-CoV-2 within the organoid cells. Taken together, we have shown that the model displays functional characteristics and cellular phenotypes found in the upper respiratory tract (BRON) and the alveolar epithelium (ALV), and that cells in organoid tissues of bronchiolar and alveolar differentiation are susceptible and permissive to infection by human respiratory viruses.

## Materials and Methods

### Cell Culture

The Madin-Darby Canine Kidney cell line, MDCK, and African green monkey kidney cell line, Vero (CCL-81, ATCC), was cultured in DMEM high glucose (Sigma-Aldrich, D5671, UK) with penicillin-streptomycin, 10% FBS and 2 mM L-glutamine. All cell lines were cultured in a humidified 37°C incubator containing 5% CO_2_, 5% O_2_.

### Human Tissue Specimens

Prior to the collection of surgical resection specimens (lobectomies, n = 8), a written informed consent was obtained in line with the requirements from the regional ethical committee (REK Vest, approval #66610). Organoids (BRON) were successfully generated from 7/8 surgical resection specimens, and differentiation into BRON and ALV were done for two of the specimens (L1 and L2). Specimens allocated to this study were carefully resected to isolate a benign part, as far as possible from the malignant lesion. The lung tissue sample was transported to the biosafety level 2 laboratory on ice in AdDF+++ medium (Advanced DMEM/F12) (Gibco, 12634028, USA), and samples were processed further within 30 min.

### Tissue Processing for Adult Stem-Cell Derived Organoids

Establishment of the airway and lung organoids was based on the protocol described by Sachs et al., (Sachs et al., 2019) with minor modifications (Hoareau et al., 2021). Briefly, the human derived lung tissue was minced using a pair of surgical scalpels (Swann-Morton, 0507, UK), lifted with a cell lifter (VWR,76036-004, USA) into a 50 ml falcon tube and washed with 10 ml AdDF+++ (Gibco, 12634028, USA) supplemented with 10 mM HEPES (Gibco, 15630106, USA), penicillin-streptomycin and 1x GlutaMax (Gibco, 35050061, China) followed by centrifugation at 400 x g for 5 min. The minced lung tissue was resuspended in AdDF+++ containing 2 mg/ml collagenase (Sigma-Aldrich, C9407, USA) and placed on an orbital shaker at 200 rpm, at 37°C for 2.5 h. After digestion, the tissue was sheared with a 10 ml glass Pasteur pipette and strained using a pre-wet 100 µm filter (Corning, 431752, USA). The strained suspension was collected and 2% FBS was added, followed by 400 x g centrifugation for 5 min. 10 ml of AdDF+++ was then added to the pellet before centrifugation. The pellet was resuspended in 2 ml of red blood cell lysis buffer (Roche, 11814389001, Germany) for 5 min at room temperature (RT) to lyse residual erythrocytes, followed by addition of 10 ml AdDF+++ and finally centrifugation at 400 x g for 5 min.

### Organoid Expansion and Differentiation

Pellets from human derived airways and lung cells, were resuspended on ice in cold Growth Factor Reduced Matrigel (Corning, #356231, USA) and 50 µl drops of Matrigel-cell suspension were distributed on pre-warmed 24-well plates (SARSTEDT, #83.3922, Germany) at 37°C for 30 min. The plate was inverted during the 30 min incubation in order to prevent organoids from sinking to the bottom. After Matrigel solidification, 400-500 µl of airway organoid alveolar (AO-A) or airway organoid bronchiolar (AO-B) medium (**Table 1**) was added to each 24-well. *CHIR99021 was only added in the AO-A medium as described (Hoareau et al., 2021). The plates were placed in a humidified 5% CO_2_, 5% O_2_ incubator set at 37°C. AO-A medium was used for alveolar differentiation of the organoids, and AO-B medium was used for bronchiolar differentiation. To ensure optimal growth conditions, AO-A and AO-B medium was changed every 4 days, where approximately 2/3 of medium was removed and 400 µl fresh AO medium was added. For freezing, the serum-free cell freezing medium, Bambanker direct (Genetics, BB01, USA), was used.

### Passaging of Organoids

Organoids were passaged every 2-3 weeks. Briefly, 1 ml of cold AdDF+++ washing medium (AdDF+++ supplemented with 1x glutamax, 10mM HEPES and 1x pen-strep) was added to each well in order to dissolve the Matrigel. Additional 1 ml of cold AdDF+++ washing medium was added to the same well, followed by vigorously pipetting up and down. The organoid suspension was centrifuged at 400 x g for 5 min at 20°C and resuspended in 2 ml TrypLE (Gibco, 12604013, Denmark) for 1-5 min at 37°C to allow organoid dissociation into single cells. Additional 10 ml of AdDF+++ was added followed by centrifugation with 400 x g for 5 min at 20°C. The organoids were resuspended in cold Growth Factor Reduced Matrigel and distributed on pre-warmed 24-well plates (SARSTEDT, #83.3922, Germany), as previously described above, in a 1:5-1:6 ratio. The ROCK inhibitor, Y-27632, was added in the media after the organoids were passaged and removed from the media after 4 days.

### IncuCyte Time-Lapse Microscopy and Quantification of Organoid Growth

A 24-well plate (SARSTEDT, #83.3922) containing organoids embedded in Matrigel was placed into an IncuCyte ZOOM microscope (Essen BioScience, Inc, USA), with a 10x objective. Images were recorded every 2 h for a total of 14 days, in a 37°C, 5% CO_2_ and 20% O_2_ incubator. To capture the images, a CCD camera with Sony ICX285 CCD sensor (Basler scout, Germany) was used. For quantification of organoid growth, the diameter of the organoids was measured from images obtained every other day, using the software ImageJ. The average of 10 organoids was used to assess organoid growth. Quantification of organoid size from H&E stained images was performed using the built-in measuring tool in ImageJ (Schneider et al., 2012). In order to make the quantification procedure of H&E sections (**Figure 1D**) comparable to the IncuCyte quantification protocol (applied for **Figures 1B, C**), the ten largest organoids were selected for quantification from each image.

### Electron Microscopy: TEM

Organoids were harvested from Matrigel using 2 ml cold AdDF+++ washing medium, washed twice with Phosphate-buffered saline (PBS), and fixed using 0.1 M Cacodylate Buffer with 2.5% glutaraldehyde (Sigma-Aldrich, G5882, USA). Specimens were embedded in resin, according to standard protocols. Briefly, samples were post-fixated with 1% Osmium tetroxide on ice for one h, followed by washing and dehydrating with ethanol (VWR, 20821.330, France). Then, the samples were incubated with ethanol/propylene oxide in a 1:1 ratio for one h, followed by the transfer in fresh resin and polymerization at 60°C for 48 h. The 70 nm sections were stained using saturated uranyl acetate in 50% ethanol and Reynolds lead citrate. Jeol JEM-1230 transmission electron microscope (Jeol, Tokyo, Japan) was used to obtain the electron microscopy images.

### RNA Purification and cDNA Synthesis

Pellet for RNA purification was prepared by washing organoids twice with PBS (Sigma-Aldrich, D8537, USA) in a 15 ml falcon tube and centrifugated at 400 x g for 5 min. To ensure dissociation, 2 ml TrypLE (Gibco, 12604013, USA) was added to the 15 ml falcon tube for 10 min at 37°C. 350 µl buffer RL (Norgen biotek, #17250, Canada) was then added, and the cells were lysed by vortexing for 15 sec, followed by the addition of 200 µl 96-100% ethanol. The RNA purification kit (Norgen biotek, #17250, Canada) was used to purify the RNA sample, according to the manufacturer’s instructions. Briefly, RNA was washed three times using washing solution A, and the RNA was collected using the elution solution from the RNA purification kit. Nanodrop 2000 Spectrophotometer (Thermo Fisher, USA) was used to measure the RNA concentration. RNA was stored in a -80°C freezer until cDNA synthesis using the cDNA reverse transcription Kit (Thermo Fisher, #4368814, USA), according to the manufacturer’s instructions. Briefly, RNA was mixed on ice with a master mix containing deoxynucleotide triphosphates, reverse transcriptase buffer, random primers, and MultiScribe™ Reverse Transcriptase. Mastercycler Nexus Thermal Cycler GSX1 (Eppendorf, Germany) was used to synthesize cDNA.

### Quantitative Real-Time PCR (RT-qPCR)

Initially, to detect the gene expression of cell type specific markers. TaqMan probes (**Table 2**), RNase free water, and LightCycler 480 probes master (Roche, 04887301001, Germany) were mixed with cDNA to make a final concentration of 25 ng/μl cDNA. The mixture was then transferred to a 384-well plate (Roche, 04729749001, USA) and centrifuged briefly at 201 x g for 1 min at 4°C. The LC 480 LightCycler (Roche Molecular Systems, USA) was used for the RT-qPCR analysis. Normalization was done using the eukaryotic 18S RNA (rRNA) gene as a reference gene (Applied biosystems, 4318839, UK). Probe for 18S and probe for the gene of interest was added in the same well. RT-qPCR was performed in triplicate for each cDNA sample, and the ΔΔCt was calculated using mean ΔCt of the triplicate from each passage of the ALV or BRON organoids (biological replicates) normalized to the average of the mean ΔCt from the four passages of ALV (**Figure 2C**) or BRON (**Figures 4C**, **6A** and **S1B**) organoids (as described in the figure legends). The ΔΔCt value was then converted to fold change, achieved using the formula 2^–ΔΔCt^ (Schmittgen and Livak, 2001). The mean fold change of the three technical replicates for each passage is shown as individual data points. GraphPadPrism v9 was used to calculate and display the geometric mean and 90% confidence interval from the plotted fold change values (biological replicates). The Mann-Whitney test was applied on the ΔCt values for each passage comparing ALV and BRON to test the alternative hypothesis that one distribution is stochastically greater than the other.

**Table 2:** Product information for TaqMan probes used in RT-qPCR assays.

Target gene	Assay ID	Product information
Surfactant protein C (SFTPC)	Hs00161628_m1	4331182, (Thermo Fisher, USA)
Podoplanin (PDPN)	Hs00366766_m1	4331182 (Thermo Fisher, USA)
Mucine 5AC (MUC5AC)	Hs01365616_m1	4331182 (Thermo Fisher, USA)
Forkhead box J1 (FOXJ1)	Hs00230964_m1	4331182 (Thermo Fisher, USA)
Keratin 5 (KRT5)	Hs00361185_m1	4331182 (Thermo Fisher, USA)
Secretoglobulin 3A2 (SCGB3A2)	Hs00369678_m1	4331182 (Thermo Fisher, USA)
Angiotensin-converting enzyme 2 (ACE2)	Hs01085333_m1	4331182 (Thermo Fisher, USA)
Transmembrane protease, serine 2 (TMPRSS2)	Hs01122322_m1	4331182 (Thermo Fisher, USA)

### Formalin Fixation and Paraffin Embedding (FFPE) of Organoids

Initially, 1 ml cold AdDF+++ washing medium was added to each well to dissolve the Matrigel. Organoids were centrifuged at 400 x g for 5 min in a 15 ml falcon tube, washed once with PBS and fixed at RT for 24 h using 3.7% formaldehyde (Sigma-Aldrich, 252549, USA). After fixation, organoids were spun down at 400 g for 5 min, colored with 1% aqueous methyl green (M8884, Sigma-Aldrich, USA) for 2 min, resuspended in approximately 50-100 µl pre-heated 1.5% low gelling temperature agarose (Sigma-Aldrich, A9045, St Louis, MO, USA) in Tris Buffered Saline (TBS) and transferred to a pre-heated 50 ml falcon tube. Organoids resuspended in agarose were spun down for 1 min at 400 x g before agarose solidification to ensure an even distribution at the bottom of the 50 ml falcon tube. The 50 ml falcon tube was placed at 4°C for 20-30 min to allow the agarose to solidify. The organoids embedded in agarose were transferred to a small cell-safe and biopsy capsule (Cell Path, EBE-0201-02A, UK) and stored in 70% ethanol until paraffin embedding and sectioning, according to standard procedures. Briefly, Formalin fixation and paraffin embedded (FFPE) organoids were cut by a cryotome into sections of 6 µm thickness and collected on SuperFrost+ object-glasses. The organoids were then used for Hematoxylin and Eosin (H&E) staining, alcian blue-periodic acid schiff (AB-PAS) histochemistry or immunofluorescence, as described below.

### H&E Staining

H&E staining of FFPE samples was performed to assess the histological features of the organoids. H&E staining was done according to standard protocols as previously described by Li et al., (Li et al., 2018). H&E-stained samples were scanned with a 40x objective, using the VS120 S6 Slide scanner (Olympus, Japan) and the images were taken with the color camera, pike F-505 (Allied Vision). The images were processed using the ImageJ software.

### AB-PAS (Alcian Blue-Periodic Acid Schiff) Histochemistry

AB-PAS was performed to assess the presence of mucin using Tissue-Tek Prisma. In brief, the organoids were stained with an alcian blue solution with a pH of 2.5 for 8 min, periodic acid for 10 min, followed by a 3 min rinse with water after each staining. Finally, the organoids were stained with schiff reagent for 15 min, rinsed with tap water for 5 min and stained with hematoxylin for 30 sec. The sections were cleared using xylene for 2 min. AB-PAS-stained samples were scanned with a 40x objective, using the VS120 S6 Slide scanner (Olympus, Japan), and the images were taken with the color camera, pike F-505 (Allied Vision). The images were processed using the ImageJ software.

### Immunofluorescent Staining

Initially, de-parafination using xylene (5 min + 10 min), 99% ethanol (2x 5 min), 96% ethanol (2x 5 min), 70% ethanol (5 min), 50% ethanol (5 min), ddH20 (2x 5 min), was performed. Followed by heat-induced antigen retrieval for 30 min at 95°C in DAKO Target Retrieval Solution (Agilent, S1699, USA) using Decloaking Chamber NxGen (Biocare Medical, USA). Organoid sections were then blocked in TBS-Tween containing 1% Bovine Serum Albumin (BSA) (Sigma-Aldrich, A1470, USA) for 30 min, followed by primary antibody incubation at 4°C overnight. The following day, organoid sections were washed three times in TBS-Tween and incubated with secondary antibodies for 1 h at RT. 50-100 µl of 0.25 µg/ml 4′,6-diamidino-2-phenylindole (DAPI) was used to stain nuclei for 2 min, and specimens were mounted by addition of 5-10 µl Prolong Diamond mounting medium (Thermo Fisher, P36961, USA) and 18 mm x 18mm confocal coverslips were placed on the specimens (Zeiss, Oberkochen, Germany). Product specifications, dilutions of primary and secondary antibodies can be found in **Tables 3** and **4**, respectively. Images were taken using the Axio Vert microscope (Zeiss, Germany).

**Table 3:** The product information and dilution of primary used to detect the various target epitopes in this study.

Target protein (abbreviation)	Primary antibody	Dilution	Company
Luciferase (LUC)	Anti-Firefly Luciferase antibody #ab181640	1:100	Abcam
ACE2	ACE2 (OTI1G4) #74512	1:200	Cell Signaling
Pan-cytokeratin (Pan-CK)	Pan-Ck, Cl. AE1/AE3, #M3515	1:200	Agilent
ARL13B	Rabbit anti-ARL13B #17711-1AP	1:400	Proteintech
Prosurfactant Protein C (Pro-SP-C)	Rabbit anti- proSP-C #AB3786	1:150	Chemicon
Aquaporin 5 (AQP5)	Goat anti-AQP5 #sc-9890	1:100	Santa Cruz
Mucin 5AC (MUC5AC)	Rabbit anti-MUC5AC #6119	1:400	CST
Clara cell 10 protein (CC10)	Mouse anti-CC10 (E-11) #sc-365992	1:200	Santa Cruz
Keratin 5 (K5)	Chicken anti-keratin 5 #905901	1:200	BioLegend
SARS-CoV-2 Nucleocapsid	SARS-Cov-2 nucleocapsid antibody #40143-R019	1:2000	Sino Biological
Double-stranded RNA (dsRNA)	Anti-dsRNA monoclonal antibody J2 #RNT-SCI-10010200	1:100	Jena Bioscience

**Table 4:** The product information and dilution of secondary antibodies used to detect the various target epitopes in this study.

Secondary antibody	Dilution	Company
Goat anti-Rabbit IgG, Alexa fluor 647 #A21244	1:400	Invitrogen
Goat anti-Rabbit IgG, Alexa fluor 647 #A32733	1:500	Invitrogen
Donkey anti-Goat IgG, Alexa fluor 647 #A21447	1:400	Invitrogen
Goat anti-Mouse IgG, Alexa fluor 546 #ab150169	1:500	Abcam
Goat anti-Chicken IgG, Alexa fluor 488 #112-605-167	1:400	Jackson
Goat Anti-Rabbit IgG # 4050-08	1:2500	Southern biotech

### Flow Cytometry

In brief, organoids were dissociated using TrypLE, centrifuged at 500 x g for 5 min, and resuspended in FACS buffer (PBS with 2% FBS). The fluorescein-conjugated lectins, Sambucus nigra agglutinin (SNA) (Vector Laboratories, FL-1301, USA) and Maackia Amurensis Lectin I (MAL 1) (Vector Laboratories, FL-1311, USA) were then diluted in a 1:500 and 1:100 ratio, respectively, in FACS buffer. The dissociated cells were then mixed with the lectins and incubated for 30 min in the dark on ice. The cells were washed twice with 1 ml FACS buffer, centrifuged at 500 x g for 5 min and resuspended in 500 µl FACS buffer, before the samples were run on an Accuri Flow Cytometer (BD Biosciences) and data were analyzed by Cytobank (Santa Clara, CA, USA).

### Generation of Pseudotype Influenza Virus

Lentiviral pseudotype viruses expressing heterologous glycoproteins from influenza virus (H5N1 and H7N1) was generated as described (Tsai et al., 2009; Zhou et al., 2012). Briefly, the calcium phosphate precipitation protocol was applied to transfect 293FT packaging cells (Invitrogen) with four plasmids needed to generate the pseudotyped virus: **1)** the packaging plasmid pCMV delta R8.2 to form the structure of the virion, **2)** the transfer vector pHR’-CMV-Luc (Addgene, Watertown, MA, USA) to transduce the target gene luciferase into cells, and **3)** the envelope vectors to express influenza virus strain specific hemagglutinin and neuraminidase (CMVR-HA and CMVR-NA, respectively) on the viral surface (Zhou et al., 2012). The titer of the H5N1 pseudotyped virus was measured as relative luciferase activity (RLA). Briefly, we add 50 µl of the prepared pseudotyped virus per well in 96 well plate, add 10000 MDCK cells/well, and culture for 72 h. After incubation, take off all the supernatant, lyse cells with 50 µl cell lysis buffer for 10-15 min, and add 50ul luciferase substrate per well before reading. The titer of the H5N1 pseudotyped virus from were around 1,5 to 2 *10E6 RLA per 50 µl. 33 µl of virus was added per well.

### Replication Competent Influenza A H7N1 Virus

The Influenza A/chicken/Italy/1081/1999 (H7N1) virus was propagated in MDCK cells, as described (NCBI:txid1381240). The titer of replication competent influenza A H7N1 virus was 1.08x10^8 PFU/ml. For infection studies, 33 µl of virus was added per well of organoids. If we estimate 500.000 cells per well, this yields an *estimated* MOI of 7.

### Infection of Human Organoids by Replicating Influenza A H7N1 Virus

The Matrigel was dissolved in cold AdDF+++ washing medium followed by collection of organoids by centrifugation at 400 x g for 5 min. Organoids were mechanically sheared using a glass pipette to expose the apical surface of epithelium to viral infection. Virus in AdDF+++ media was mixed with the organoids and incubated for 3 h at 37°C. After inoculation, for each condition 3 x 200 µl of organoid-virus mix was transferred to wells in 24-well ultra-low attachment plate (Corning, #3473, USA) at 37°C, 5% CO_2_, 5% O_2_ incubator for 24 h incubation. An additional 200 µl of AdDF+++ was added per well. Organoid culture supernatants were harvested 72 h post-infection and replication was assessed by plaque assay. Briefly, ~500,000 MDCK cells/well were seeded in 6-well plates the day before infection. On the day of infection, 500 µl of organoid culture supernatant was added and incubated with the MDCK cells for 45 min at 37°C. After washing, DMEM high glucose supplemented with pen-strep, 4 mM L-glutamine (Sigma-Aldrich, G-0781) and 50 mM HEPES buffer with 2 ug/ml of TPCK-trypsin was added. 1.6% pre-heated agarose was added in a 1:1 ratio and the cells were incubated at 37°C for 72 h. Cells were fixed for 24 h at RT using a final concentration of 3.7% formaldehyde. After washing, plaques were visualized by staining with 0.5% crystal violet solution.

### Infection With a Clinical Isolate of SARS-CoV-2-WA1 and Detection of Organoid Susceptibility by RT-qPCR

Bronchiolar organoids were thawed and transferred to an ultra-low attachment plate well (Corning, #3473, USA). The organoids were infected with SARS-CoV-2-WA1 (Washington strain) at an approximate multiplicity of infection (MOI) of 0.5 (assuming 300,000 cells) and cultured in AdDF+++. After 24 h, the media was changed, and the organoids were transferred to a new plate. SARS-CoV-2-WA1 infected organoids and supernatants were harvested after 96 h. Cell pellets were lysed in TRIzol and RNA was purified through phase separation. Reverse transcription was used for cDNA production (1 µg RNA per sample), followed by RT-qPCR analysis, using the SYBR green method, for the SARS-CoV-2 N gene, using GAPDH as a housekeeping gene.

### Infection of Organoid Cultures for TCID 50 Assay to Test Virus Propagation of a Clinical Isolate of SARS-CoV-2

A clinical isolate of SARS-CoV-2 (hCoV-19/Norway/Bergen-01/2020 GISAID accession number: EPI_ISL_541970) was propagated in Vero cells (CCL-81, ATCC), and the titer of the propagated virus were determined to be 1.96x10^6 TCID50/ml. To ensure that SARS-CoV-2 virus could access the lumen of the organoids, bronchiolar and alveolar organoids were initially dissociated by TrypLE and subsequently inoculated with SARS-CoV-2 virus at 3.92*10^5 and 3.92*10^3 TCID50, which equals an estimated MOIs of 0.5 and 0.005, respectively. Organoid cells were inoculated with virus for 1 h at 37°C before cells were collected by centrifugation to remove the virus containing media. The organoids were washed with fresh medium to remove any residual medium. The infected organoids were seeded in 150 µl medium in ultra-low attachment plates and cultured in humidified 37°C incubator with 5% CO2. After 72 h and 96 h, organoids were collected by centrifugation. Supernatant was collected and stored at 4°C until use.

### Quantification by TCID50 Assay With Immunostaining Against SARS-CoV-2 NP

For quantification of replicative virus by the TCID50 assay, harvested supernatant were added to Vero cells seeded in 96-well plates. For all experimental conditions, 4 biological replicates were quantified by a 10-fold dilution series á 6 steps, and 7 technical replicates were performed per biological sample. Medium harvested from un-infected organoids served as negative control, and Vero cell propagated virus stock served as positive control in this experiment. Vero cells (CCL-81, ATCC) were incubated with viral supernatant for 24 h at 37°C. Cells were fixed and permeabilized with methanol (Sigma Cat.32213-2.5L-M) and 0.6% H2O2 (Sigma Cat. H1009-100ML). Cells were then blocked in 5% skimmed milk, 1% BSA and 0.1% Tween-20 in PBS, overnight at 4 degrees, and then incubated with rabbit monoclonal IgG against SARS-CoV2 NP (Sino Biological Cat. 40143-R019-100, 1:2000 o/n at 4°C), prior to 6 times washes with 0.05% Tween-20 in PBS. Cells were then incubated (2 h, RT) with biotinylated goat anti-rabbit IgG (H+L) (Southern Biotech Cat. 4050-08). Following 6 washes, streptavidin-HRP (Southern Biotech Cat 7105-05), the reactions were developed with o-phenylenediamine dihydrochloridec (OPD) (Sigma Cat. P8287-100TAB, 1:2500). OPD were incubated for 10 min at RT in the dark, and the reaction was stopped by addition of 1N/0.5M H_2_SO_4._ Results were read at a Synergy H1 plate reader (BioTek) at 490 nm. The TCID50 was calculated using the Reed-Muench method. The lower detection limit (LOD, defined as the lowest concentration of virus that can be reliably detected in ≥95% of specimens tested were determined empirically by testing series dilutions of viral stock) were determinied empirically by testing series dilutions of viral stock. of SARS-CoV-2 (hCoV-19/Norway/Bergen-01/2020 GISAID accession number: EPI_ISL_541970) stock as a positive control in the assay. LOD for this assay was 110 TCID50/mL.

### Statistical Analysis

Data were presented as mean values, +/- standard deviations (SD), or as fold changes +/- 90% confidence interval from a representative experiment as specified on the axes. In general, statistical tests were performed using the GraphPad Prism 9 software (San Diego, CA, USA). The Mann-Whitney test was used for data analysis. The specific test is indicated in the legends for each experiment. The following symbols are given to report statistical significance: Ns *P* > 0.05, *P ≤ 0.05, **P ≤ 0.01 ***P ≤ 0.001, ****P ≤ 0.0001.

## Discussion

The frequency and severity of viral pandemics are expected to increase as the global population increases and areas become more densely populated (Arif and Sengupta, 2020; Whiting, 2020). Therefore, it is of uttermost importance that we learn from the ongoing pandemic and improve global pandemic preparedness. Respiratory viruses are the most common acute infections (Boncristiani et al., 2009). Human respiratory virus belongs to several virus families that initially target airway epithelial cells, and it may be difficult to distinguish causative virus based on the clinical symptoms. Respiratory virus usually transmit *via* airway secretions from an infected individual, and may spread *via* direct or indirect contact, or *via* droplet and aerosol transmission (Siegel et al., 2007). Viral infections confined to the upper respiratory tract usually cause mild symptoms. When viral infections spread from the upper respiratory tract to the lungs it may cause severe morbidity and mortality. It is important to understand the modes of transmission, the viral entry, the regional specific virus-host interactions, and to gain a more complete understanding of how the virus mediates disease. The availability of improved and well-characterized pre-clinical models that resemble the various parts of the human respiratory airways and lungs are an important, as these models provide a superior tool to study virus-host interactions, test prophylactics and therapeutics for existing and emerging viruses, and to support the study of co-infections. More widespread application of these improved models in virology is expected to aid in the development of prophylactic measures as well as therapeutic options. This knowledge will allow a more rapid response when novel virus strains emerge. Here, we present a reproducible and representative adult stem cell derived model for virology that faithfully recapitulates the regional hallmarks of both bronchiolar and alveolar epithelium, and we show that the model is well suited to support viral infection and replication of selected respiratory viruses.

Murine models have been vital to study the *in vivo* replication of virus and the interaction of virus with the animal host defense system. However, significant differences between the murine and human immune systems may limit the value of their findings and mice lack appropriate receptors for SARS CoV-2. A recent study described use of transgenic mice with ectopic expression of human ACE2 to detect SARS CoV-2 antigens in bronchial epithelial cells, macrophages and alveolar epithelial cells (Bao et al., 2020). Further, the humanized mouse strain with induced human ACE2 expression, K18-hACE2 mice from Jackson labs, (McCray Jr et al., 2007) were shown to support SARS-CoV-2 replication in respiratory and brain tissues, and the authors showed that the model recapitulate many of the findings observed in patients with COVID-19 including changes in the expression of pro-inflammatory molecules similar to what have been observed in patients with COVID-19 post infection. A publication by Yinda and colleagues (Yinda et al., 2021) later showed that the rapid inflammatory response and observed pathology observed in this model bears resemblance to COVID-19. As the authors also point out, it should be noted that the murine K18-hACE2 model relies on artificial ectopic expression of hACE2 under the widely expressed Keratin 18 promotor, and this facilitates an expression pattern of hACE2 that does not precisely recapitulate the expression pattern in humans (Yinda et al., 2021).

There is no doubt that these mice are useful models to assess vaccines and antiviral agents against COVID-19, however, the limitation introduced by exogenous viral receptor expression remains, and may affect the tropism as well as replication kinetics. Due to the phylogenetic proximity to humans, non-human primate models have previously been utilized to study SARS and MERS (Haagmans et al., 2004; Wang et al., 2017; Chandrashekar et al., 2020; Munster et al., 2020; Rockx et al., 2020). During the current COVID-19 pandemic, there has been a significant increase in the demand and there is currently a large shortage of non-human primates (Zhang, 2020). SARS-CoV-2 infection were shown to induce protective immunity against re-challenge in rhesus macaques (Chandrashekar et al., 2020). However, the non-human primate models experience only the moderate disease and not the severe ARDS sometimes observed in humans (Munster et al., 2020).

Three dimensional (3D) organotypic models, or organoids, can be established from either induced pluripotent stem cells (iPSCs) or adult stem cells. Organoid cultures of human airway and lung tissue serve as a more relevant model of the heterogeneity and complexity of the human respiratory epithelium, compared to homogenous 2D cell cultures of immortalized animal cell lines or malignant human cell lines which are often not susceptible to multiple types of virus infection, and thus unsuited to study the pathogeneses caused by co-infections. Other research groups have pioneered the organoid technology, establishing organoids of bronchiolar differentiation from adult stem cells (Zhou et al., 2018; Sachs et al., 2019). We showed here that organoids displaying proper differentiation of epithelium characteristic of the conducting airways and the respiratory epithelium with respect to histo-architecture and cellular composition could be rapidly established from adult stem cells using the BRON protocol established by Hans Clevers laboratory (Sachs et al., 2019), and recently optimized by us for efficient differentiation into both bronchiolar and alveolar differentiation (Hoareau et al., 2021). This study shows that the organoids retain characteristic histological features and the expected cellular composition of the two epithelia throughout 4 passages. The ability for early differentiation as well as long-term expansion both represent huge experimental advantages. Patient-derived organoids have been found to retain similar frequencies of basal, club, multi-ciliated and secretory cells for over 1 year in culture in other studies (Sachs et al., 2019). Long term culture in combination with longitudinal sampling in this adapted model of BRON and ALV cultures would be required in this model also to validate if the robustness of the model is comparable to this. The fact that organoids recapitulate essential functional aspects of the lungs are of great importance as it contributes to a more complex and dynamic culturing system. In contrast, the frequently applied 2D cell cultures are easy to work with, but they do not recapitulate this functional complexity.

The alveoli are the functional units of the lung and consist mainly of AT1 cells, which are large and flat squamous cells that have an important role in gas exchange. In fact, 95% of the gas exchange surface of the lung is comprised of AT1 cells (Wu and Tang, 2021). In contrast, AT2 cells are smaller cells of a cuboidal shape. AT2 cells synthesize lung surfactant, which is composed of 90% lipids and helps to reduce the surface tension during breathing movements (Andreeva et al., 2007). Highly specialized organelles, termed lamellar bodies, are found in differentiated AT2 cells, and serves to package the surfactant (Sever et al., 2021). In addition, AT2 cells have been found to play a vital role in re-establishing homeostasis after lung damage. Under these challenging conditions, AT2 cells have been shown to display stem cell potential and the ability of AT2 cells to self-renew and differentiate into AT1 cells has been shown in several studies (Barkauskas et al., 2013; Desai et al., 2014; Katsura et al., 2019). However, it is still unknown whether all or just a subset of AT2 cells harbor this stem cell potential. In the alveolar organoids, we determined the presence of alveolar epithelium cells, Type 1 pneumocytes and Type 2 pneumocytes (AT1 and AT2 cells, respectively). AT1 cell studies have been restricted due to a lack of genes with expression unique to AT1 cells. PDPN was utilized as a AT1 cell marker in this study in order to be able to compare the expression levels with a previous report (Hoareau et al., 2021). However, PDPN is also expressed in basal cells (Van de Laar et al., 2014), among other cell types from other tissues, and it is therefore not an AT1 cell specific marker. Furthermore, the higher expression of KRT5 in the alveolar organoids compared to bronchiolar organoids requires further investigation as it calls the derivation of AT2 cells into question and suggests expansion of the niche-independent stem cells, namely basal cells. Nevertheless, KRT5 and PDPN expression profiles are not similar, suggesting a more complicated expression panel by different cell subtypes. The differentiation of alveolospheres with functional AT1 and AT2 cells have been a longstanding challenge in the field, and we acknowledge that there is still room for improvement in the differentiation protocols in order to generate a more robust ALV model.

The aim of this study was to determine whether the organotypic model described is suitable for infection by respiratory viruses. Influenza viruses are amongst the most common causes of human respiratory infections and are single-stranded, enveloped, negative-sense RNA viruses that belong to the *Orthomyxoviridae* family (Piret and Boivin, 2021). The Influenza A virus is responsible for most of the disease burden in humans, and its structural proteins include nucleoprotein, two matrix-proteins in addition to the two surface glycoproteins neuraminidase (NA) and hemagglutinin (HA) (Webster et al., 1992). A major concern with influenza viruses is their ability to undergo antigenic drift and subsequently acquire mutations in NA and HA that allows them to escape pre-existing immunity. Human influenza viruses preferentially bind to sialic acids (SA) attached to galactose *via* an α 2,6 linkage (Rogers et al., 1983; Connor et al., 1994), and these are known to be ubiquitously expressed in the human respiratory epithelium. As expected, high expression of SA and galactose were detected in both bronchiolar and alveolar organoids in this study. We further demonstrated the bronchiolar and alveolar organoids were susceptible to infection with pseudotype influenza H5N1 and H7N1 viruses and support active replication of influenza A H7N1 virus. Influenza virus infection has also been shown in human adult stem cells-derived airway organoids (Zhou et al., 2018).

SARS-CoV-2 preferably enters cells through binding *via* its Spike-protein (S-protein) to the angiotensin-converting enzyme 2 (ACE2) (Hoffmann et al., 2020). The receptor, ACE2, has been found to be expressed in organs such as the kidney, heart, testis, intestines and the lungs (Harmer et al., 2002). ACE2 expression has been shown to determine the host susceptibility to the SARS-CoV-2 (Lanjanian et al., 2021). However, the ACE2 protein expression is either low or lacking in the majority of cells found in the conducting and respiratory epithelium (Hikmet et al., 2020).

Artificial expression of human ACE2 is required to make human 2D cell lines susceptible to SARS-CoV-2 infection, such in the case of the human adenocarcinoma cell line A549. In contrast, VeroE6 cells, derived from African green monkey, and the human lung cancer cell line, Calu-3, endogenously express ACE2 (Ren et al., 2006). However, VeroE6 cells are not derived from humans and the cell line does not represent primary human airway cells, where expression was found to be either low or lacking in the majority of cells (Hikmet et al., 2020). In contrast to cell lines and animal models that depend on ectopic ACE2 expression to make them SARS-CoV-2 susceptible, we detected endogenous ACE2 expression in the alveolar and bronchiolar organoids using RT-qPCR and immunofluorescence. Heterogenous organoids with endogenous physiologically relevant levels of ACE2 expression may be more well suited to study viral tropism and particularly useful in studies of alternative modes of entry and alternative entry factors (van der Vaart and Clevers, 2021). ACE2 protein has been found to be heterogeneously expressed in the human respiratory tract, and in line with this, we observed a co-localization of ciliated cells and ACE2, which is consistent with previous reports (Lee et al., 2020; Mulay et al., 2020). The ACE2 expression in ciliated cells was very strong compared to the neighboring cells. SARS-CoV-2 has been found to predominantly infect ciliated cells, in models such as ALI culture systems and reconstructed human airway epithelium model (Mulay et al., 2020; Robinot et al., 2021). However, SARS-CoV-2 infection in mucus-secreting goblet cells, using 2D cultures, has not been observed in other studies (Hou et al., 2020; Lamers et al., 2020). From this we know that much is still to be learned regarding the tropism and replication kinetics of SARS-CoV-2 in human primary cells. This knowledge is particularly important for optimal rational selection and testing of potential antiviral drugs, and we envision that the organoid models will greatly facilitate this research in the future. Variation in ACE2 expression profiles also exists between individuals. In particular, ACE2 is found to be upregulated in the small airway epithelia of smokers (Zhang et al., 2020). Organoids derived from patients with co-morbidities and various ACE2 expression profiles, or *in vitro* simulation of these conditions, could serve as a valuable platform to study the effect on susceptibility, and this serves to be explored further.

In addition to ACE2, the serine protease, TMPRSS2, is important for ACE2 host entry as it cleaves the viral S-protein and consequently promotes SARS-CoV-2 cell entry at the plasma membrane (Hoffmann et al., 2020; Zang et al., 2020). We detected TMPRSS2 expression in alveolar and bronchiolar organoids. Our findings are consistent with previous studies, where in addition to TMPRSS2, TRMPRSS4 and TMPRSS11D expression was also detected (Zhou et al., 2018). In contrast, most 2D models have a low serine protease activity and require the addition of trypsin treated TPCK for the cleavage of viral glycoprotein HA to subsequently allow Influenza A virus to infect efficiently. Trypsin has been found to interfere with host pathogen-defense mechanisms, where in particular interferon signaling in MDCK cells was strongly inhibited in the presence of trypsin (Seitz et al., 2012). Therefore, organoid models that do not require exogenous trypsin may be more suitable for interferon-studies. The lack of TMPRSS2 could also indicate that these frequently applied cell lines are infected through viral entry *via* the endocytic pathway, and not by ACE2 and TMPRSS2 dependent cleavage at the cell membrane that is the preferred entry pathway in human primary respiratory epithelium (van der Vaart and Clevers, 2021). Thus, highlights yet another vital difference between traditionally used virology models and the human respiratory epithelium (Bohan et al., 2021; van der Vaart and Clevers, 2021). Furthermore, the endosomal cysteine protease, cathepsin L, has also been found to be involved in SARS-CoV-2 entry through the endosomal route (Padmanabhan et al., 2020), but *via* a different pathway. Having confirmed endogenous expression of ACE2 and TMPRSS2 in both bronchiolar and alveolar organoids, we aimed to evaluate infection of the organoids by a clinical SARS-CoV-2 isolate, Washington strain. Successful infection of the organoid cultures was confirmed using RT-qPCR to detect the presence of SARS-CoV-2 N gene mRNA in infected organoids of bronchiolar as well as alveolar differentiation. Next, we aimed to explore whether the infected organoids were permissive to SARS-CoV-2 *i.e.* if they were able to produce infectious viral particles. Of note, so called abortive infection, *i.e.* infection without successful replication, was recently shown for SARS-CoV infected macrophages (Yilla et al., 2005) and dendritic cells (Law et al., 2005). Despite abortive infection, the SARS-CoV infection were able to induce expression of proinflammatory, but not antiviral cytokine production in the infected macrophages (Cheung et al., 2005; Tseng et al., 2005). For human respiratory epithelium, the innate recognition mechanisms and response to viral infection is being explored at the single cell level, and there is still an urgent need to identify biomarkers that can predict which patients will experience a severe disease course upon infection (Abassi et al., 2020; Yamada et al., 2021). By TCID50 assays we were able to show that a clinical isolate of SARS-CoV-2 (hCoV-19/Norway/Bergen-01/2020), could efficiently replicate in organoids of bronchiolar as well as alveolar differentiation. Virus production was detected in organoids infected at an estimated MOI of 0.5 at both 72h and 96h post-infection. Dysmorphic cells of SARS-CoV-2 infected human lungs were characterized by the frequent appearance of syncytia, characterized by several nuclei with an ample cytoplasm surrounded by a single plasma membrane (Bussani et al., 2020). Histopathological examination of the infected organoids in this study also revealed the resemblance to the human COVID19 lungs with the presence of dysmorphic pneumocytes and prominent syncytia formation, indicating viral infection. Immunostaining to detect the SARS-CoV-2 Nucleocapsid Protein (NP) is one of the core components of the SARS-CoV-2, and the NP oligomerizes to form the closed capsule protecting the viral RNA. Immunofluorescent staining of the infected organoid cultures of bronchiolar and alveolar differentiation in this study confirmed successful infection of SARS-CoV-2 (hCoV-19/Norway/Bergen-01/2020).

Much remains to be learnt about the immunological outcome of human cell type specific infection, and the respiratory airway and lung organoid model presented in this study may serve as a useful tool to learn gain insight into the cell type specific kinetics of viral infection and replication, and the initial communication from infected cells to the innate immune cells. This is important as more simplistic models, such as permissive cell lines, although more robust and easier to infect, cannot be applied to address remaining questions related to cell type specific and regional differences in the viral life cycle and the immune interactions. Due to the physiologically relevant heterogeneity of the pHAE and organoid models, their application is particularly useful in combination with spatially resolved single cell high-dimensional methodologies like mass cytometry (CyToF), imaging mass cytometry (IMC) or single-cell RNA seq. Furthermore, compared to cell line-based models, organoid models require careful validation of differentiation status and expression of viral entry factors prior to use, which in turn encourage the development of true interdisciplinary collaborations.

In conclusion, we have developed and characterized an organoid model from adult stem cells with efficient bronchiolar as well as alveolar differentiation, characterized the presence of the various cell types expected in the differentiated human tissues *in situ*, as well as the endogenous expression of viral entry factors required for respiratory influenza and SARS-CoV-2 viruses. The particular strength of this model is that bronchiolospheres and alveolospheres may be generated efficiently from a single tissue specimen, and the organoids represent the histoarchitecture and the cell types representative of the upper and lower respiratory epithelium, respectively. Successful infection in the model were confirmed by two clinical isolates of SARS-CoV-2 (WA-1 and hCoV-19/Norway/Bergen-01/2020), and successful replication of SARS-CoV-2 in the organoids of bronchiolar and alveolar differentiation were determined by TCID50 assays. Thus, our findings strongly support application of the model in future studies of viral tropism and replication kinetics, as well as screening of antiviral compounds in available *human* pre-clinical organoid models. If successfully implemented, the knowledge and application of human organoid models in the study of existing and emerging respiratory viruses are important to reduce the impact of future pandemics.

## Data Availability Statement

The original contributions presented in the study are included in the article/**Supplementary Material**. Further inquiries can be directed to the corresponding author.

## Ethics Statement

The studies involving human participants were reviewed and approved by Regional Ethics Committee Vest (REK Vest), Vestland, Norway. The patients/participants provided their written informed consent to participate in this study.

## Author Contributions

All authors contributed to the conception and design of experiments. Performed the experiments: CE, FZ, DB, ML, LH, NLu, AE. Analyzed the data: CE, FZ, DB, MR, GR, NLu, LA, NL, RC, WM, LS, JL, and AE. Contributed materials/reagents/ analysis tools: MR, MA, FG, PS, MB; TH, HR, LA. Wrote the first draft of the paper: CE, ML, LS, JL, AE. All authors were involved in article revision and approved the submitted version.

## Funding

This study was partly supported by the Research Council of Norway through its Centres of Excellence funding scheme, project number 223250 (CCBIO affiliates). The Influenza Centre is funded by the University of Bergen, Ministry of Health and Care Services, Helse Vest (F-11628), the Trond Mohn Foundation (TMS2020TMT05), the European Union (EU IMI115672 FLUCOP, H2020 874866 INCENTIVE, H2020 101037867 VACCELERATE, EU IMI101007799 Inno4Vac) and Nanomedicines Flunanoair (ERA-NETet EuroNanoMed2, JTC2016), and the Research Council of Norway GLOBVAC program (284930). WM was supported by NIH/NIAID grant R01 AI134733 and a contract from BerGenBio ASA. DB was supported by NIH/NIAID grant T32 AI007511. The funders were not involved in the study design, collection, analysis, interpretation of data, the writing of this article, or the decision to submit it for publication.

## Conflict of Interest

The authors declare that the research was conducted in the absence of any commercial or financial relationships that could be construed as a potential conflict of interest.

## Publisher’s Note

All claims expressed in this article are solely those of the authors and do not necessarily represent those of their affiliated organizations, or those of the publisher, the editors and the reviewers. Any product that may be evaluated in this article, or claim that may be made by its manufacturer, is not guaranteed or endorsed by the publisher.

## Abbreviations

AB-PAS, Alcian Blue Periodic Acid–Schiff; ACE2, Angiotensin-converting enzyme 2; AdDF+++, Advanced DMEM/F-12; ARDS, Acute Respiratory Distress Syndrome; AT1, Type 1 pneumocytes; AT2, Type 2 pneumocytes; BSA, Bovine Serum Albumin; CoV, Coronavirus; COVID-19, Coronavirus Disease-2019; DAPI, 4′,6-diamidino-2-phenylindole; ECM, Extracellular matrix; EGF, Epidermal growth factor; FBS, Fetal Bovine Serum; FFPE, Formalin-fixed paraffin-embedded; FOXJ-1, Forkhead box J1; H&E, Hematoxylin and Eosin; HA, Hemagglutinin; HCQ, Hydroxychloroquine; iPSC, Induced pluripotent stem cells; KRT5, Keratin 5; LB, Lysogeny broth; MAL I, Maackia Amurensis Lectin I; MAPK, Mitogen-activated protein kinase; MERS, Middle East Respiratory Syndrome; MERS-CoV, Middle East Respiratory Syndrome Coronavirus; MOI, Multiplicity of infection; NA, Neuraminidase; NHP, Non-Human-Primate; PBS, Phosphate Buffered Saline; pHAE, primary human airway epithelial cells; PDPN, Podoplanin; RT-qPCR, Quantitative real-time reverse transcription polymerase chain reaction; RT, Room temperature; SA, Sialic acid; SARS, Severe Acute Respiratory Syndrome; SARS-CoV-2, Severe Acute Respiratory Syndrome Coronavirus 2; SCGB, Secretoglobins; SFTPC, Surfactant protein C; SNA, Sambucus nigra agglutinin; S-protein, Spike-protein; TEM, Transmission Electron Microscopy; TGF- β, Transforming growth factor beta; TBS, Tris Buffered Saline; TMPRSS2, Transmembrane protease, serine 2; WHO, World Health Organization.
