# Supplementary material for: Human Organotypic Airway and Lung Organoid Cells of Bronchiolar and Alveolar Differentiation Are Permissive to Infection by Influenza and SARS-CoV-2 Respiratory Virus
Source: Front Cell Infect Microbiol. 2022 Mar 14;12:841447. doi: 10.3389/fcimb.2022.841447 (PMC8964279; doi:10.3389/fcimb.2022.841447)
Supplement: Supplementary file 1 [file DataSheet_1.pdf]

Supplementary Material

Human organotypic airway and lung organoid cells of bronchiolar and alveolar differentiation are permissive to infection by influenza and SARS-CoV-2 respiratory virus

Camilla Tvedt Ekanger[1,2,3], Fan Zhou[3], Dana Bohan[4], Maria Lie Lotsberg[1,2], Maria Ramnefjell[2, 5], Laurence Hoareau[6,9], Gro Vatne Røsland[1,2], Ning Lu[1,2], Marianne Aanerud[6,7], Fabian Gärtner [6,7], Pirjo Riitta Salminen[6,8], Mariann Bentsen[6,9], Thomas Halvorsen [6,9], Helge Ræder [6,9], Lars A. Akslen[2,5], Nina Langeland[6,10], Rebecca Cox[3,6,11], Wendy Maury[4], Linda Elin Birkhaug Stuhr[1], James B. Lorens[1,2], and Agnete S. T. Engelsen[1,2*]

1 Department of Biomedicine, Faculty of Medicine, University of Bergen, Bergen, Norway

2 Centre for Cancer Biomarkers CCBIO, Department of Clinical Medicine, University of Bergen, Bergen, Norway

3 The Influenza Centre, Department of Clinical Science, University of Bergen, Bergen, Norway

4 Department of Microbiology and Immunology, University of Iowa, Iowa City, IA, United States

5 Department of Pathology, Haukeland University Hospital, Bergen, Norway

6 Department of Clinical Science, Faculty of Medicine, University of Bergen, Bergen, Norway

7 Department of Thoracic Medicine, Haukeland University Hospital, Bergen, Norway

8 Section of Cardiothoracic Surgery, Department of Heart Disease, Haukeland University Hospital, Bergen, Norway

9 Department of Pediatrics, Haukeland University Hospital, Bergen, Norway

10 Department of Medicine, Haukeland University Hospital, Bergen, Norway

11 Department of Microbiology, Haukeland University Hospital, Bergen, Norway

*** Correspondence:**
Dr. Agnete S. T. Engelsen
E-mail: agnete.engelsen@uib.no

RESULTS

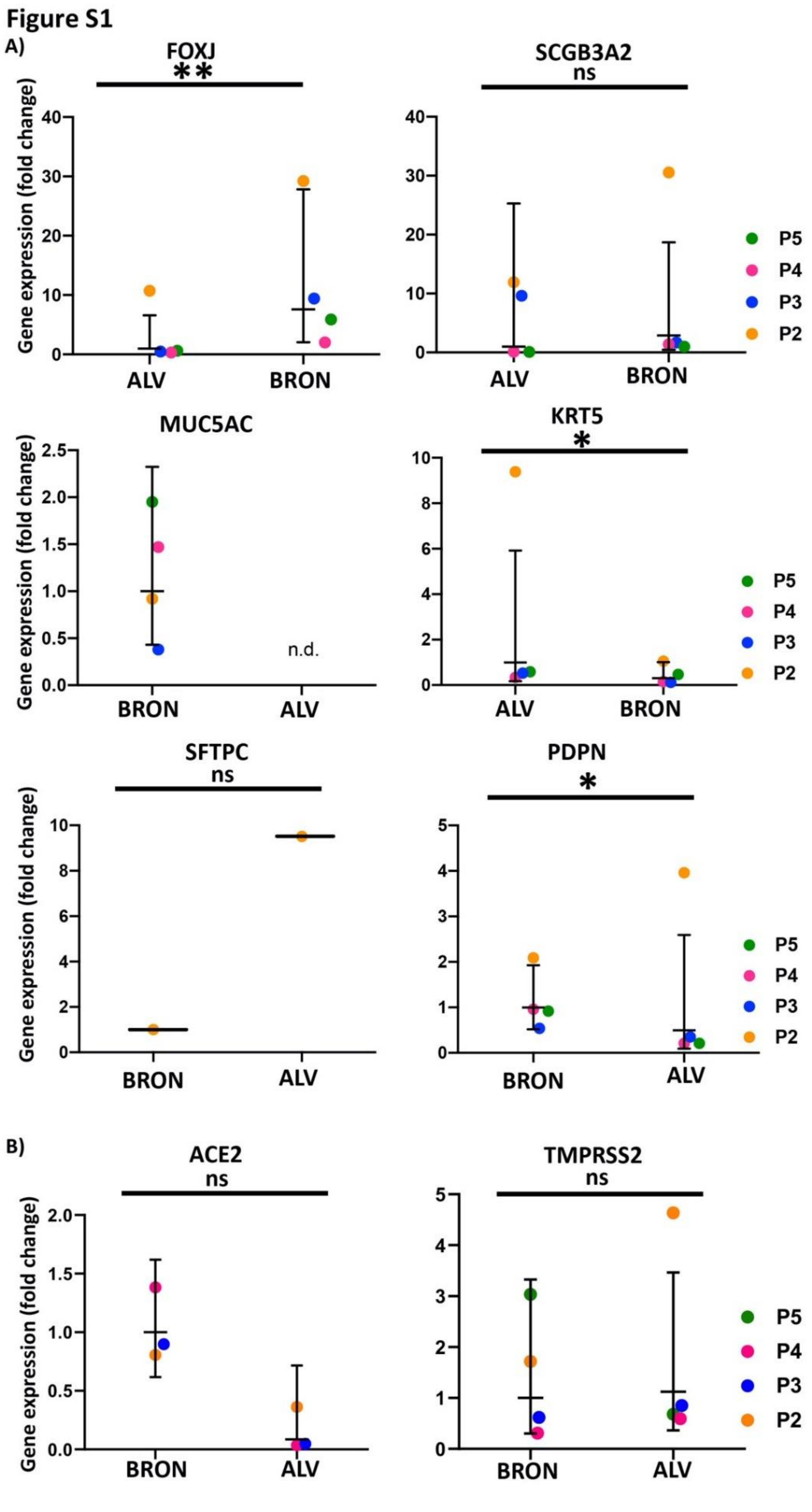
Figure S1
A)
FOXJ
**
SCGB3A2
ns
Gene expression (fold change)
40
30
20
10
0
ALV
BRON
P5
P4
P3
P2
MUC5AC
KRT5
*
2.5
2.0
1.5
1.0
0.5
0.0
n.d.
10
8
6
4
2
SFTPC
ns
PDPN
*
5
4
3
2
1
B)
ACE2
ns
TMPRSS2
ns

FIGURE S1

A) Relative expression of the cell-type markers, FOXJ1 (ciliated cell), SCGB3A2 (club cell), MUC5AC (goblet cell), and KRT5 (basal cell), SFTPC (AT2 cell), and PDPN (AT1 cell). Relative quantification was performed using the Livac ($2^{-\Delta\Delta Ct}$) method. Bronchiolar (BRON) and alveolar (ALV) are normalized against the average delta Ct of BRON organoid for the markers, PDPN, SFTPC and MUC5AC. BRON and ALV organoids are normalized against the average delta Ct of alveolar organoids for the markers, FOXJ, SCGB3A2 and KRT5. BRON and ALV organoids have been passaged 4 times (P2-P5) in the respective differentiation media. The mean value of three technical replicates for each passage are shown here as individual points. Mean represents the 2^(mean (-ΔΔCT)) of the biological replicates harvested at different passages. GraphPadPrism v9 was used to calculate and display the geometric mean and 90% confidence interval of the biological replicates (passages). Statistics were performed on the delta Ct values using the Mann-Whitney test. ns = not significant. A significant difference in gene expression were detected for FOXJ (***P* = 0,0034), KRT5 (**P* = 0,0387) and PDPN (* *P* = 0,0387) between ALV and BRON organoids. The gene expression profile shown here are from patient L1.

B) ACE2 (left) and TMPRSS2 (right) expression is detected in patient L1 organoids of bronchiolar (BRON) and alveolar (ALV) differentiation from passage 2-5. Relative quantification was performed using the Livac method and alveolar organoids are normalized to the average delta Ct of bronchiolar organoids. The mean value of three technical replicates for each passage are shown as individual data points. GraphPadPrism v9 was used to calculate and display the geometric mean and 90% confidence interval of the biological replicates (passages). Statistics were performed on the delta Ct values using the Mann-Whitney test. No statistically significant differences were observed in the median delta Ct values of ACE2 and TMPRSS2 between BRON and ALV cultures (ns = not significant).

VIDEO S1:

IncuCyte timelapse video showing the organoid formation in alveolar (ALV) differentiation media. Weblink: http://vedlegg.uib.no/?id=bc53ca0f221f160089306c24c67cf6fd

VIDEO S2:

IncuCyte timelapse video showing the organoid formation in bronchiolar (BRON) differentiation media. Weblink: http://vedlegg.uib.no/?id=16670b783bb9633fd600b4c1ec23fba8 (BRON)

VIDEO S3:

Video show beating cilia in organoids of bronchiolar (BRON) differentiation medium. Weblink: http://vedlegg.uib.no/?id=dee2d0b60466c9a50761d96ee067e72b
